# Phytoliths in selected broad-leaved trees in China

**DOI:** 10.1038/s41598-020-72547-w

**Published:** 2020-09-23

**Authors:** Yong Ge, Houyuan Lu, Can Wang, Xing Gao

**Affiliations:** 1 Key Laboratory of Vertebrate Evolution and Human Origins, Institute of Vertebrate Paleontology and Paleoanthropology, Chinese Academy of Sciences, Beijing, 100044 China; 2 Key Laboratory of Cenozoic Geology and Environment, Institute of Geology and Geophysics, Chinese Academy of Sciences, Beijing, 100029 China; 3 Center for Excellence in Life and Paleoenvironment, Chinese Academy of Sciences, Beijing, 100044 China; 4 Center for Excellence in Tibetan Plateau Earth Science, Chinese Academy of Sciences, Beijing, 100101 China; 5 University of Chinese Academy of Sciences, Beijing, 100049 China; 6 Department of Archaeology, School of History and Culture, Shandong University, Jinan, 250100 China

**Keywords:** Plant sciences, Geology, Palaeontology, Palaeoecology

## Abstract

Broad-leaved trees are widely distributed from tropical to temperate zones in China, reference collections of phytoliths from these taxa are crucial for the precise reconstruction of paleoenvironments and the study of early plant resource exploitation. However, not much has been published on the phytoliths produced by modern broad-leaved trees. In this study, we collected samples of 110 species that cover the common species distributed in Northern and Southern China, and extracted phytoliths from leaves, twigs and fruits, in order to investigate the phytoliths types and production in these species. We found that only 58 species were phytoliths producers, and that 23 distinct phytoliths morphotypes could be recognized. The results showed that phytoliths types and production in Northern and Southern China could be similar in the two regions. Through analyzing previously published data and our data, Elongate brachiate geniculate, Polygonal tabular, Elongate facetate, Tracheary annulate/facetate geniculate and Tracheary annulate/facetate claviform have been proposed to be the potential diagnostic types for broad-leaved trees in general. This study provided a preliminary reference of phytoliths in modern broad-leaved trees, and could be used in the identification of phytoliths in sediments and archaeological contexts.

## Introduction

Phytoliths are micro silica bodies produced by plants from silica deposits made in and around the cells^1^. As phytoliths maintain the shape of the cells and tissue in which they are formed, phytoliths can be taxonomically significant^1–3^. Compared with other plant micro-remains, phytoliths can especially reveal information about Poaceae species, as Poaceae plants produce more phytoliths than most other taxa^4,5^, and phytoliths can be preserved in sediments where organic material (such as pollen or seed) is typically not well preserved, such as in fire pits (where materials were directly burnt) and highly oxidized soils^4–6^. Thus, phytolith analysis has been a valuable tool for researchers.

Phytoliths are considered to reflect local vegetation due to their in situ deposition^7^, reference collections of regional scale^8–16^ and certain taxa^17–23^ have been shown to be useful for geological and archaeological studies^2,3,24–32^. In recent years, phytolith analysis has helped researchers make much progress in understanding vegetation change in paleoecology^33–35^, the reconstruction of paleoclimate^36,37^ and the exploitation of plant resources in the early stages of agriculture^38–44^. However, as woody plants typically have shown a comparatively low degree of silicification^4,5,45^, phytoliths in broad-leaved trees have not been extensively studied. While a few phytolith studies involving species of broad-leaved trees from tropical areas and other regions have been conducted^12,15,46–54^, little phytolith research has been conducted on woody taxa from sub-tropical and temperate China^4,55,56^.

Previous studies commonly illustrated the morphology of phytoliths observed in the leaves of broad-leaved trees by SEM^45,46,50,51^ or light microscope^12,15,48,49,52,55–58^. Some studies also revealed that spherical and elongate types of phytoliths could be found in the stem^59^, wood and bark^60^ in some woody plants. However, although many studies provided the morphology of phytoliths observed in broad-leaved trees, there has not been a reliable identification criterion, especially in temperate China. The illustration of phytoliths in broad-leaved trees sometimes was used as identification criteria^4^, however, no systematic comparison has been made. Thus, the identification of phytoliths from broad-leaved trees in sediments was difficult in practice, which hindered the precise reconstruction of the paleoenvironment and the understanding of woody plant utilization by the ancestors.

To solve the issues on phytoliths in broad-leaved trees, we selected specimens that cover the taxa of common broad-leaved trees in temperate China to carry out the phytolith analysis. In this study, we provided the phytoliths morphology of common broad-leaved trees in China, and several morphotypes were proposed to be potentially diagnostic for the identification of broad-leaved trees in general. Our results could be a valuable tool for the identification of phytoliths both in natural and archaeological sediments, especially in temperate zones that covered by broad-leaved trees.

## Material and methods

A total of 110 species, belonging to 33 families Table 1 were collected for analysis. These species were collected from four regions, Changbai Mountain (N 41°40′, E 125°45′), Gongga Mountain (N 30°02′, E 101°57′), Beijing Botanical Garden (N 40°10′, E 116°12′), and Xiamen Botanical Garden (N 24°27′, E 118°05′), during August to October, in the years 2001, 2004, 2015 and 2019, respectively. To investigate the phytolith types and frequencies in these species, the leaves, branchs and fruit were separately treated using a modified wet oxidation method^61^. Every part (leaf, twig and fruit) of each specimen was cleaned with distilled water in an ultrasonic water bath to remove adhering particles and then dried in an air drying box for 24 h, the dried materials (mostly 5 g, the species with large leaves were used one whole leaf), were cut into smaller parts and placed in separate tubes and the tubes filled with 20 ml (or enough to submerge the materials) saturated nitric acid and left for one night; the next day the tubes with materials were heated in a water bath (at 90 °C) for at least 2 h, then the solutions were centrifuged at 3000 rpm for 10 min. After removing the supernatant, 5 to 10 ml (or enough to submerge the materials) perchloric acid was added to each tube and then heated in the water bath until the solution became clear and transparent; then the solutions were centrifuged and rinsed with distilled water 3 times and then with ethyl alcohol for a last rinse. Then, 3 ml of ethyl alcohol was added into each tube, and mixed using a Vortex Mixers for 30 s to make the residues homogenous. One drop of the mixture from each tube was mounted on separate slides using Canada Balsam for further observation.

**Table 1:** Information of the studied specimens.

Family	Latin name	Phytolith production index^a^	Tree/shrub	Parts for experiment	Sampling site
Aceraceae	*Acer caudatum* Wall	A	Tree	Leaf and twig	Gongga Mountain
Aceraceae	*Acer komarovii* Pojark	A	Tree	Leaf and twig and fruit	Changbai Mountain
Aceraceae	*Acer laxiflorum* Pax	A	Tree	Leaf and twig	Gongga Mountain
Aceraceae	*Acer mandshuricum* Maxim	A	Tree	Leaf and twig and fruit	Changbai Mountain
Aceraceae	*Acer negundo* Linn	A	Tree	Leaf and twig and fruit	Changbai Mountain
Aceraceae	*Acer oliverianum* Pax	A	Tree	Leaf and twig	Gongga Mountain
Aceraceae	*Acer tataricum* sub *ginnala* (Maximowicz) Wesmael	A	Shrub/tree	Leaf and twig	Changbai Mountain
Aceraceae	*Acer ukurunduense* Trautv. et Mey	A	Tree	Leaf and twig and fruit	Changbai Mountain
Actinidiaceae	*Clematoclethra scandens* Maxim	NP	Vine	Leaf and twig	Gongga Mountain
Anacardiaceae	*Rhus chinensis* Mill	A	Shrub/tree	Leaf and twig	Gongga Mountain
Anacardiaceae	*Rhus potaninii* Maxim	C	Tree	Leaf and twig	Gongga Mountain
Anacardiaceae	*Rhus punjabensis* Stewart var. *sinica* (Diels) Rehd.et Wils	NP	Tree/shrub	Leaf and twig	Gongga Mountain
Araliaceae	*Eleutherococcus senticosus* (Ruprecht & Maximowicz) Maximowicz	NP	Shrub	Leaf and twig and fruit	Changbai Mountain
Araliaceae	*Gamblea ciliata* C. B. Clarke var. *evodiifolia* (Franchet) C. B. Shang et al	NP	Shrub/tree	Leaf and twig	Gongga Mountain
Asteraceae	*Myripnois dioica* Bunge	NP	Shrub	Leaf	Gongga Mountain
Berbeidaceae	*Berberis poiretii* Schneid	R	Shrub	Leaf and twig	Changbai Mountain
Berberidaceae	*Berberis diaphana* Maxin	A	Shrub	Leaf and twig	Gongga Mountain
Berberidaceae	*Berberis dictyophylla* Franch	A	Shrub	Leaf and twig	Gongga Mountain
Berberidaceae	*Mahonia bealei* (Fort.) Carr	A	Shrub/tree	Leaf and twig	Gongga Mountain
Betulaceae	*Betula delavayi* Franch	A	Tree/shrub	Leaf and twig	Gongga Mountain
Betulaceae	*Corylus heterophylla* Fisch. ex Trautv	A	Shrub/tree	Leaf and twig and fruit	Changbai Mountain
Betulaceae	*Corylus mandshurica* Maxim	A	Shrub	Leaf and twig	Changbai Mountain
Caprifoliaceae	*Lonicera prostrata* Rehder	NP	Shrub	Leaf and twig	Gongga Mountain
Caprifoliaceae	*Lonicera trichosantha* Bureau & Franchet	NP	Shrub	Leaf and twig	Gongga Mountain
Caprifoliaceae	*Sambucus adnata* Wall. ex DC	A	Under shrub	Leaf and twig	Gongga Mountain
Caprifoliaceae	*Viburnum betulifolium* Batal	NP	Shrub/tree	Leaf and twig	Gongga Mountain
Caprifoliaceae	*Viburnum foetidum* Wall. var. *ceanothoides* (C. H. Wright) Hand.-Mazz	A	Shrub/tree	Leaf and twig	Gongga Mountain
Caprifoliaceae	*Viburnum opulus* L. var. *calvescens* (Rehd.)	NP	Shrub	Leaf and twig and fruit	Changbai Mountain
Caprifoliaceae	*Viburnum* sp.	A	Shrub/tree	Leaf and twig	Gongga Mountain
Celastraceae	*Euonymus chuii* Hand.-Mazz	NP	Shrub	Leaf and twig	Gongga Mountain
Celastraceae	*Euonymus phellomanus* Loesener	NP	Shrub	Leaf and twig and fruit	Changbai Mountain
Celastraceae	*Euonymus szechuanensis* C. H. Wang	NP	Shrub	Leaf and twig	Gongga Mountain
Cornaceae	*Cornus controversa* Hemsley	A	Tree	Leaf and twig and fruit	Changbai Mountain
Cornaceae	*Cornus hemsleyi* C. K. Schneider & Wangerin	A	Shrub/tree	Leaf and twig	Gongga Mountain
Cornaceae	*Cornus schindleri* subsp. *poliophylla* (C. K. Schneider & Wangerin) Q. Y. Xiang	A	Shrub/tree	Leaf and twig	Gongga Mountain
Cornaceae	*Cornus schindleri* Wangerin	NP	Shrub/tree	Leaf and twig	Gongga Mountain
Ericaceae	*Rhododendron calophytum* Franch	A	Shrub/tree	Leaf and twig	Gongga Mountain
Ericaceae	*Rhododendron concinnum* Hemsl	A	Shrub	Leaf and twig	Gongga Mountain
Ericaceae	*Rhododendron galactinum* Balf.f. ex Tagg	NP	Shrub/tree	Leaf and twig	Gongga Mountain
Ericaceae	*Rhododendron intricatum* Franch	NP	Shrub	Leaf and twig	Gongga Mountain
Ericaceae	*Rhododendron rubiginosum* Franch	A	Shrub	Leaf and twig	Gongga Mountain
Ericaceae	*Rhododendron strigillosum* Franch	A	Shrub	Leaf and twig	Gongga Mountain
Ericaceae	*Rhododendron tatsienense* Franch	NP	Shrub	Leaf and twig	Gongga Mountain
Ericaceae	*Rhododendron vernicosum* Franch	NP	Shrub/tree	Leaf and twig	Gongga Mountain
Euphorbiaceae	*Aleurites moluccana* (L.) Willd	A	Tree	Leaf and twig and fruit	Gongga Mountain
Euphorbiaceae	*Discocleidion rufescens* (Franch.) Pax & K. Hoffm	NP	Tree/shrub	Leaf and twig	Gongga Mountain
Euphorbiaceae	*Flueggea suffruticosa* (Pall.) Baill	A	Shrub	Leaf and twig and fruit	Changbai Mountain
Euphorbiaceae	*Leptopus chinensis* (Bunge) Pojark	U	Shrub	Leaf and twig	Gongga Mountain
Eupteleaceae	*Euptelea pleiosperma* J. D. Hooker & Thomson	A	Shrub/tree	Leaf and twig	Gongga Mountain
Fagaceae	*Fagus engleriana* Seem	A	Tree	Leaf	Gongga Mountain
Fagaceae	*Quercus acutissima* Carr	A	Tree	Leaf	Gongga Mountain
Fagaceae	*Quercus mongolica* Fischer ex Ledebour	A	Tree	Leaf and twig	Changbai Mountain
Ginkgoaceae	*Ginkgo biloba* Linn	NP	Tree	Leaf and twig	Beijing
Ginkgoaceae	*Ginkgo biloba* Linn	NP	Tree	Leaf and twig	Fujian
Hamamelidaceae	*Corylopsis willmottiae* Rehd. & E. H. Wils	NP	Shrub/tree	Leaf and twig	Gongga Mountain
Hamamelidaceae	*Hamamelis mollis* Oliv	NP	Shrub/tree	Leaf and twig	Gongga Mountain
Hippocastanaceae	*Aesculus chinensis* Bunge	A	Tree	Leaf	Gongga Mountain
Juglandaceae	*Pterocarya hupehensis* Skan	A	Tree	Leaf and twig	Gongga Mountain
Lauraceae	*Machilus microcarpa* Hemsl	A	Tree	Leaf and twig	Gongga Mountain
Leguminosae	*Lespedeza bicolor* Turcz	A	Shrub	Leaf and twig	Changbai Mountain
Leguminosae	*Lespedeza cuneata* (Dumont de Courset) G. Don	A	Shrub	Leaf and twig	Gongga Mountain
Liliaceae	*Smilax* sp.	A	Shrub	Leaf and twig	Gongga Mountain
Magnoliaceae	*Oyama sieboldii* (K. Koch) N. H. Xia & C. Y. Wu	A	Tree	Leaf and twig	Changbai Mountain
Moraceae	*Ficus tikoua* Bur	A	Vine	Leaf and vine	Gongga Mountain
Moraceae	*Morus australis* Poir	A	Shrub/tree	Leaf and twig	Gongga Mountain
Pittosporaceae	*Pittosporum truncatum* Pritz	A	Shrub	Leaf and twig	Gongga Mountain
Rhamnaceae	*Rhamnus parvifolia* Bunge	NP	Shrub	Leaf and twig and fruit	Changbai Mountain
Rosaceae	*Cerasus maximowiczii* (Rupr.) Kom	A	Tree	Leaf and twig	Changbai Mountain
Rosaceae	*Cerasus* sp.	NP	Shrub	Leaf and twig	Gongga Mountain
Rosaceae	*Cotoneaster divaricatus* Rehder & E. H. Wilson	NP	Shrub	Leaf and twig	Gongga Mountain
Rosaceae	*Potentilla fruticosa* L	NP	Shrub	Leaf and twig	Gongga Mountain
Rosaceae	*Pyracantha crenulata* (D. Don) Roem	NP	Shrub/tree	Leaf and twig	Gongga Mountain
Rosaceae	*Rosa acicularis* Lindl	A	Shrub	Leaf and twig and fruit	Changbai Mountain
Rosaceae	*Rosa helenae* Rehder & E. H. Wilson	A	Shrub	Leaf and twig	Gongga Mountain
Rosaceae	*Rosa murielae* Rehder & E. H. Wilson	A	Shrub	Leaf and twig	Gongga Mountain
Rosaceae	*Rubus amabilis* Focke	NP	Shrub	Leaf and twig	Gongga Mountain
Rosaceae	*Rubus biflorus* Buch.-Ham. ex Sm	NP	Shrub	Leaf and twig	Gongga Mountain
Rosaceae	*Rubus crataegifolius* Bunge	NP	Shrub	Leaf and twig	Changbai Mountain
Rosaceae	*Rubus inopertus* (Focke) Focke	NP	Shrub	Leaf and twig	Gongga Mountain
Rosaceae	*Rubus lambertianus* Ser. var. *glaber* Hemsl	NP	Shrub	Leaf and twig	Gongga Mountain
Rosaceae	*Rubus macilentus* Cambess	NP	Shrub	Leaf and twig	Gongga Mountain
Rosaceae	*Rubus niveus* Thunb	NP	Shrub	Leaf and twig	Gongga Mountain
Rosaceae	*Rubus rosifolius* Smith	NP	Shrub	Leaf and twig	Gongga Mountain
Rosaceae	*Rubus setchuenensis* Bureau & Franch	NP	Shrub	Leaf and twig	Gongga Mountain
Rosaceae	*Rubus subtibetanus* Hand.-Mazz	NP	Shrub	Leaf and twig	Gongga Mountain
Rosaceae	*Sorbaria sorbifolia* (Linn.) A. Br	A	Shrub	Leaf and twig	Changbai Mountain
Rosaceae	*Sorbus multijuga* Koehne	A	Shrub/tree	Leaf and twig	Gongga Mountain
Rosaceae	*Sorbus oligodonta* (Cardot) Hand.-Mazz	NP	Tree	Leaf and twig	Gongga Mountain
Rosaceae	*Sorbus prattii* Koehne	NP	Shrub	Leaf and twig	Gongga Mountain
Rosaceae	*Sorbus setschwanensis* (C. K. Schneid.) Koehne	NP	Shrub	Leaf and twig	Gongga Mountain
Rosaceae	*Spiraea longigemmis* Maxim	A	Shrub	Leaf and twig	Gongga Mountain
Rosaceae	*Spiraea ovalis* Rehder	NP	Shrub	Leaf and twig	Gongga Mountain
Rutaceae	*Phellodendron amurense* Rupr	A	Tree	Leaf and twig	Changbai Mountain
Salicaceae	*Populus lasiocarpa* Oliv	A	Tree	Leaf and twig	Changbai Mountain
Salicaceae	*Populus* sp.	A	Tree	Leaf and twig	Beijing
Salicaceae	*Salix dissa* C. K. Schneid	NP	Shrub	Leaf and twig	Gongga Mountain
Salicaceae	*Salix ernestii* C. K. Schneid	C	Shrub	Leaf and twig	Gongga Mountain
Salicaceae	*Salix hylonoma* var. *liocarpa* (Goerz) G. Zhu	NP	Tree	Leaf and twig	Gongga Mountain
Salicaceae	*Salix rehderiana* C. K. Schneid	NP	Shrub/tree	Leaf and twig	Gongga Mountain
Salicaceae	*Salix wallichiana* Andersson	NP	Shrub/tree	Leaf and twig	Gongga Mountain
Saxifragaceae	*Philadelphus schrenkii* Rupr	A	Shrub	Leaf and twig and fruit	Changbai Mountain
Saxifragaceae	*Ribes himalense* Royle ex Decne	NP	Shrub	Leaf and twig	Gongga Mountain
Saxifragaceae	*Ribes longiracemosum* Franch	NP	Shrub	Leaf and twig	Gongga Mountain
Saxifragaceae	*Ribes moupinense* Franch	NP	Shrub	Leaf and twig	Gongga Mountain
Schisandraceae	*Schisandra chinensis* (Turcz.) Baill	NP	Vine	Leaf and twig and fruit	Changbai Mountain
Scrophulariaceae	*Paulownia fargesii* Franch	A	Tree	Leaf and twig	Gongga Mountain
Stachyuraceae	*Stachyurus chinensis* Franch	NP	Shrub	Leaf and twig	Gongga Mountain
Staphyleaceae	*Tapiscia sinensis* Oliv	NP	Tree	Leaf and twig	Gongga Mountain
Tiliaceae	*Tilia mandshurica* Rupr. et Maxim	A	Tree	Leaf and twig	Changbai Mountain
Ulmaceae	*Zelkova schneideriana* Hand.-Mazz	U	Tree	Leaf and twig	Gongga Mountain

^a^Phytolith production index refer to the result part, which *NP *non producer, *A *abundant, *C *common, *U *uncommon.

Analyses of the phytoliths thus extracted were conducted under a Leica DM 750 microscope at 400 × magnification. For phytolith identification and counting a total of 100 fields (10 × 10, evenly distributed) under the microscope was analyzed on each slide. If no phytoliths were observed in a slide after scanning the whole slide, then another slide was prepared and scanned, and a replica using more dried materials was conducted for a final examination. After observing all the slides, representative phytolith types were chosen to provide photographic images. All morphotypes are described using the International Code for Phytolith Nomenclature 2.0 (ICPN 2.0)^62^.

The Principal Components Analysis (PCA analysis) was conducted in C2 program^63^ to study the relationship between phytoliths types and studied species. The Mann–Whitney *U* test was conducted in R software^64^ to find out the significance of differences between phytoliths type and production in species from southern and northern China.

## Results

### Phytoliths types in the studied species

A total of 23 different types of phytoliths were observed in the studied species. Typical phytoliths types are shown in Figs. 1 and 2, and more detailed illustrations of phytoliths produced by each specimen can be found in the Supplementary Figures 1–13. Phytoliths types are described in Table 2.

**Figure 1:**
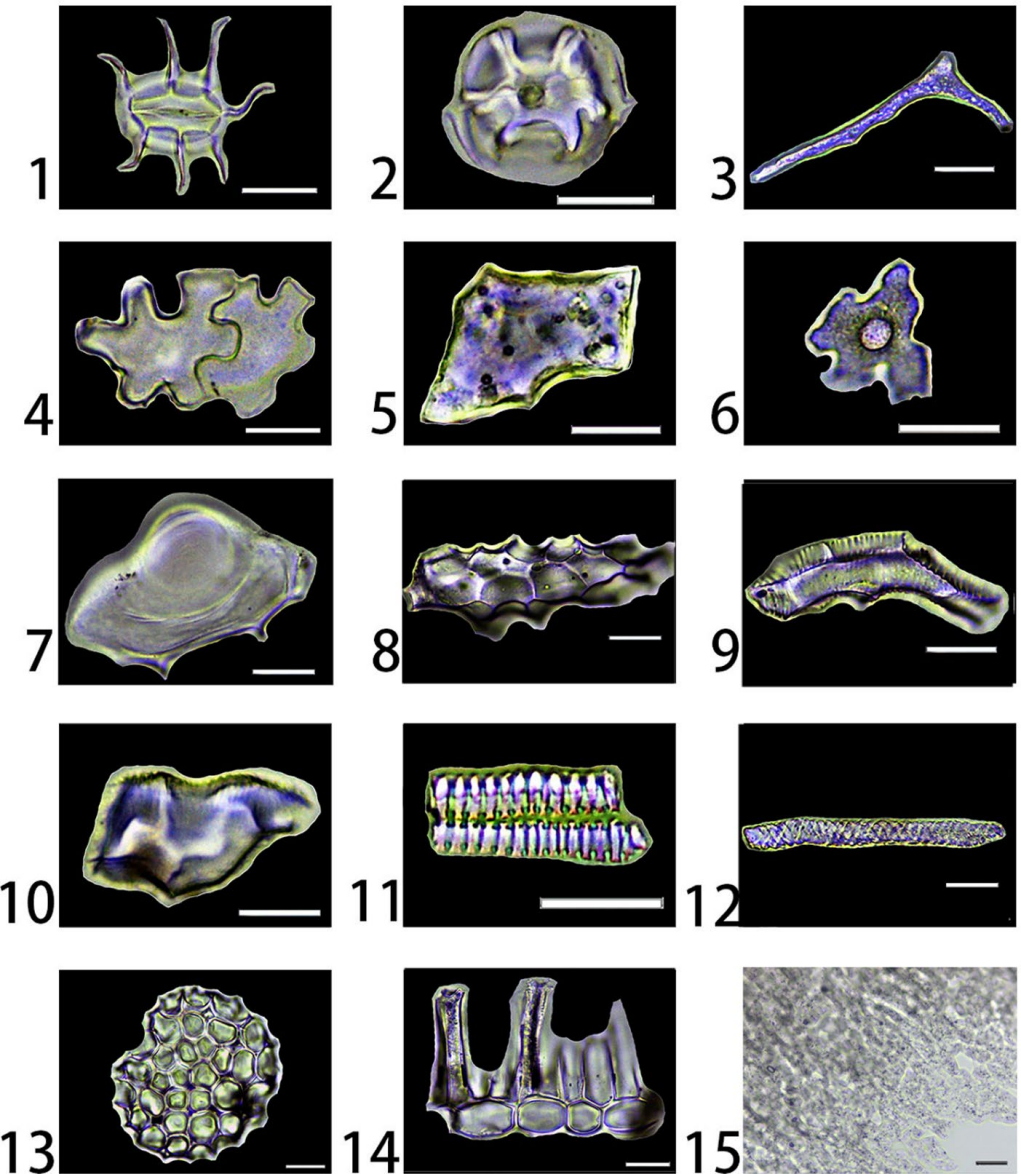
Phytoliths types observed in this study: 1–2. Stomate stellate (*Paulownia fargesii* and *Mahonia bealei*, leaf); 3. Elongate brachiate geniculate (*Quercus mongolica*, leaf); 4. Irregular sinuate (*Lespedeza bicolor*, leaf); 5. Polygonal tabular (*Paulownia fargesii*, leaf); 6. Trichome irregular tubercule (*Cornus schindleri* sub *poliophylla*, leaf); 7. Trichome bulbous irregular (*Smilax* sp., leaf); 8. Elongate facetate (*Pittosporum truncatum*, leaf); 9. Tracheary annulate/facetate geniculate (*Pittosporum truncatum*, leaf); 10. Tracheary annulate/facetate claviform (*Oyama sieboldii*, leaf); 11. Tracheary annulate (*Rhus potaninii*, leaf); 12. Tracheary helical (*Mahonia bealei*, leaf); 13. Spheroid favose (*Cornus* c*ontroversa*, leaf); 14. Elongate entire and Spheriod hollow (*Acer oliverianum*, leaf), they are often found articulate; 15. Irregular articulated granulate (*Aleurites moluccana*, fruit husk). Scale bars are 20 μm.

**Figure 2:**
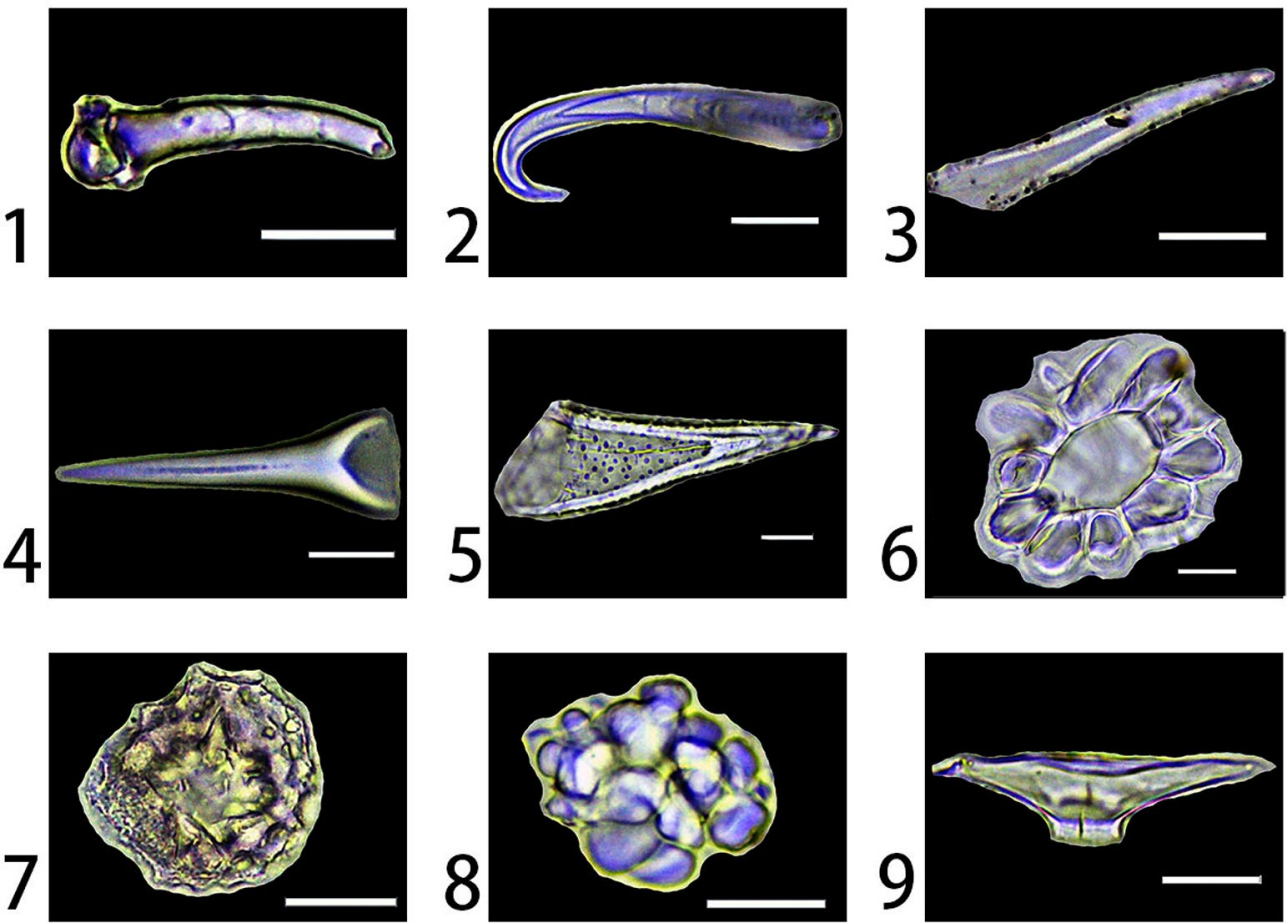
Phytoliths types observed in this study: 1. Acute bulbosus (*Rosa helenae*, leaf); 2. Acute uncinate (*Smilax* sp., leaf); 3. Acute (*Leptopus chinensis*, leaf); 4. Acute acicular (*Morus australis*, leaf); 5. Acute echinate (*Ficus tikoua*, leaf); 6. Hair base (*Acer komarovii*, leaf); 7. Trichome spheroid plicate/cavate (*Euptelea pleiosperma*, leaf); 8. Ellipsoidal nodulate ( *Populus* sp., leaf); 9. Trichome fusiform cavate ( *Cornus controversa*, leaf). Scale bars are 20 μm.

**Table 2:** Phytoliths types observed in this study.

Phytolith morphotype	Description	Produced in species	Categories	See figure
Stomate stellate	Originating from silicified stomata cells, usually having oblong bodies and often having filiform protuberances along two sides	*Acer caudatum, Acer komarovii, Acer laxiflorum, Acer mandshuricum*, *Acer negundo*, *Acer oliverianum*, *Acer tataricum* sub *ginnala, Acer ukurunduense*, *Rhus chinensis*, *Berberis diaphana*, *Berberis dictyophylla*, *Mahonia bealei*, *Betula delavayi*, *Corylus heterophylla*, *Corylus mandshurica*, *Sambucus adnata*, *Viburnum foetidum* var. *ceanothoides*, *Viburnum* sp., *Cornus controversa*, *Cornus hemsleyi*, *Cornus schindleri* sub *poliophylla*, *Rhododendron calophytum*, *Rhododendron concinnum*, *Rhododendron rubiginosum*, *Aleurites moluccana*, *Leptopus chinensis*, *Fagus engleriana*, *Quercus acutissima*, *Quercus mongolica*, *Aesculus chinensis*, *Lespedeza bicolor*, *Lespedeza cuneata*, *Smilax* sp., *Oyama sieboldii*, *Pittosporum truncatum*, *Cerasus maximowiczii*, *Rosa acicularis*, *Rosa helenae*, *Sorbaria sorbifolia*, *Sorbus multijuga*, *Spiraea longigemmis*, *Populus lasiocarpa*, *Populus* sp., *Salix ernestii*, *Paulownia fargesii*	Stomata class	Figure 1-1 and 1-2 Supplementary Figure 1-I-a, 1-II-a, 1-III-a, 2-I-a, 2-II-a, 2-III-a, 2-IV-a, 3-I-a, 3-II-d, 3-IV-a, 3-V-a, 4-I-a, 4-II-a, 4-III-a, 4-IV-a, 5-I-a, 5-II-a, 5-III-a, 5-IV-a, 6-I-a, 6-II-a, 6-III-a, 6-IV-a, 7-I-a, 7-II-a, 7-IV-a, 8-II-a, 8-III-a, 8-IV-a, 9-I-a, 9-Iv-a, 10-I-a, 10-II-a, 10-III-a, 11-II-a, 11-III-a, 11-IV-a, 11-V-a, 12-I-a, 12-II-a, 12-II-a, 12-V-a, 12-VI-a, 13-I-a, 13-III-a
Elongate brachiate geniculate	Possibly originating from silicified sclerenchyma, often bent and branched to form a “Y” shape	*Rhododendron calophytum*, *Quercus mongolica*, *Machilus microcarpa*	Silicified cell class	Figure 1-3 Supplementary Figure 6-III-d, 8-IV-f, 9-III-c
Irregular sinuate	Originating from silicified epidermal cells, have irregular margins and often found articulated, sometimes a conical protuberance form at the center	*Acer komarovii*, *Corylus heterophylla*, *Corylus mandshurica*, *Fagus engleriana*, *Pterocarya hupehensis*, *Lespedeza bicolor*, *Pittosporum truncatum*, *Phellodendron amurense*, *Philadelphus schrenkii*	Silicified cell class	Figure 1-4 Supplementary Figure 1-II-c, 4-III-b, 4-IV-b, 8-II-b, 9-II-b, 9-IV-b/c, 11-II-b, 12-IV-a, 13-II-a
Polygonal tabular	Originating from silicified epidermal cells have polygonal margins and flat surfaces	*Acer caudatum, Acer laxiflorum*, *Acer mandshuricum*, *Acer negundo*, *Acer oliverianum*, *Acer tataricum* sub *ginnala, Acer ukurunduense*, *Rhus chinensis*, *Rhus potaninii*, *Berberis diaphana*, *Berberis dictyophylla*, *Mahonia bealei*, *Betula delavayi*, *Corylus heterophylla*, *Corylus mandshurica*, *Sambucus adnata*, *Viburnum* sp., *Cornus controversa*, *Cornus hemsleyi*, *Cornus schindleri* sub *poliophylla*, *Rhododendron calophytum*, *Rhododendron concinnum*, *Rhododendron rubiginosum*, *Aleurites moluccana*, *Flueggea suffruticosa*, *Euptelea pleiosperma*, *Quercus acutissima*, *Quercus mongolica*, *Aesculus chinensis*, *Lespedeza bicolor*, *Lespedeza cuneata*, *Smilax* sp., *Ficus tikoua*, *Cerasus maximowiczii*, *Rosa acicularis*, *Rosa helenae*, *Sorbus multijuga*, *Populus lasiocarpa*, *Salix ernestii*, *Paulownia fargesii*, *Tilia mandshurica*	Silicified cell class	Figure 1-5 Supplementary Figure 1-I-b/c, 1-III-b/c, 2-I-b/c, 2-II-b, 2-III-b/c, 2-IV-d, 3-I-b, 3-II-a, 3-III-a/b, 3-IV-b, 3-V-b, 4-I-b, 4-II-c/e, 4-III-c, 4-IV-c, 5-I-c, 5-III-b/c, 5-IV-b, 6-I-b, 6-II-b, 6-III-c, 6-IV-c, 7-I-c, 7-II-b, 7-III-b, 8-I-a, 8-III-b, 8-IV-b, 9-I-c, 9-IV-d, 10-I-b, 10-II-b, 10-IV-a, 11-III-b, 11-IV-b, 11-V-d, 12-II-b/c, 12-V-b, 13-I-a, 13-III-b, 13-IV-a,
Trichome irregular tubercule	Originating from silicified epidermal trichome elements, have irregular margins and a tubercule on the surface, with a granular rather than smooth surface texture	*Cornus schindleri* sub *poliophylla*	Hair tissue class	Figure 1-6 Supplementary Figure 6-II-d/f
Trichome bulbous irregular	Originating from silicified epidermal trichome elements, have irregular margins and often articulated, a bulbous protuberance may be found in the center	*Smilax* sp.	Hair tissue class	Figure 1-7 Supplementary Figure 10-II-c
Elongate facetate	Originating from silicified tracheid tissues, the width of the short axis can be over 20 microns, the surface of the bodies has several flat to slightly concave areas	*Machilus microcarpa*, *Pittosporum truncatum*	Tracheid/vascular tissue class	Figure 1-8 Supplementary Figure 9-III-a, 11-II-f
Tracheary annulate/facetate geniculate	Originating from silicified tracheid tissues, the width of the short axis can be around 20 microns, can be slightly bent, have several flat to slightly concave areas on one side of the surface and an annulate texture on the other side	*Pittosporum truncatum*	Tracheid/vascular tissue class	Figure 1-9 Supplementary Figure 11-II-e
Tracheary annulate/facetate claviform	Originating from silicified tracheid tissues, have a claviform shape with several flat to slightly concave areas on one side and an annulate texture on the other side	*Machilus microcarpa*, *Oyama sieboldii*	Tracheid/vascular tissue class	Figure 1-10 Supplementary Figure 9-III-b, 10-III-b
Tracheary annulate	Originating from silicified vascular tissues, have elongate bodies with annulate texture	*Acer caudatum, Acer komarovii*, *Acer laxiflorum*, *Acer mandshuricum*, *Acer negundo*, *Acer oliverianum*, *Acer tataricum* sub *ginnala, Acer ukurunduense*, *Rhus chinensis*, *Rhus potaninii*, *Berberis diaphana*, *Berberis dictyophylla*, *Betula delavayi*, *Corylus heterophylla*, *Corylus mandshurica*, *Sambucus adnata*, *Viburnum* sp., *Cornus controversa*, *Cornus hemsleyi*, *Aleurites moluccana*, *Flueggea suffruticosa*, *Leptopus chinensis*, *Euptelea pleiosperma*, *Fagus engleriana*, *Quercus acutissima*, *Quercus mongolica*, *Aesculus chinensis*, *Pterocarya hupehensis*, *Machilus microcarpa*, *Smilax* sp., *Oyama sieboldii*, *Cerasus maximowiczii*, *Rosa helenae*, *Sorbaria sorbifolia*, *Spiraea longigemmis*, *Phellodendron amurense*, *Populus lasiocarpa*, *Populus* sp., *Salix ernestii*, *Philadelphus schrenkii*, *Paulownia fargesii*, *Tilia mandshurica*	Tracheid/vascular tissue class	Figure 1-11 Supplementary Figure 1-I-e, 1-II-e, 1-III-e, 2-I-e, 2-II-d, 2-III-f, 2-IV-e, 3-I-e, 3-II-c, 3-III-b/c, 3-IV-c, 3-V-c, 4-II-d, 4-III-e, 4-IV-f/g, 5-I-g, 5-III-e, 5-IV-e, 6-I-e, 7-II-f, 7-III-c, 7-IV-c, 8-I-c, 8-II-d, 8-III-e, 8-IV-e, 9-I-g, 9-II-d, 9-III-d, 10-II-g, 10-III-c, 11-III-c, 11-V-c, 12-I-c, 12-III-c, 12-IV-d, 12-V-c, 12-VI-c, 13-I-c, 13-II-c, 13-III-d, 13-IV-c,
Tracheary helical	Originating from silicified vascular tissues, has an elongate body with helical texture on the surface	*Mahonia bealei*, *Lespedeza bicolor*	Tracheid/vascular tissue class	Figure 1-12 Supplementary Figure 4-I-c, 9-IV-f
Spheroid favose	Possibly originating from silicified mesophyll cells, has a spheroid to ellipsoid shape with multiple hollowed holes on it	*Acer caudatum, Acer komarovii*, *Acer laxiflorum*, *Acer mandshuricum*, *Acer negundo*, *Acer oliverianum*, *Acer ukurunduense*, *Rhus chinensis*, *Corylus heterophylla*, *Corylus mandshurica*, *Sambucus adnata*, *Viburnum foetidum* var. *ceanothoides*, *Viburnum* sp., *Cornus controversa*, *Rhododendron calophytum*, *Rhododendron concinnum*, *Rhododendron rubiginosum*, *Aleurites moluccana*, *Flueggea suffruticosa*, *Euptelea pleiosperma*, *Fagus engleriana*, *Quercus mongolica*, *Aesculus chinensis*, *Lespedeza cuneata*, *Smilax* sp., *Pittosporum truncatum*, *Rosa acicularis*, *Phellodendron amurense*, *Populus lasiocarpa*, *Salix ernestii*, *Philadelphus schrenkii*, *Paulownia fargesii*	Silicified cell class	Figure 1-13 Supplementary Figure 1-I-d, 1-II-d, 1-III-d, 2-I-d, 2-II-c, 2-III-e, 2-IV-b, 3-I-d, 3-II-c, 4-III-b, 4-IV-c/d, 5-I-f, 5-II-b, 5-III-d, 5-IV-c, 6-I-c, 6-II-c, 6-III-b, 6-IV-b, 7-I-d, 7-II-c, 7-III-a, 8-I-b, 8-II-c, 8-IV-c, 9-I-d, 10-I-c, 10-II-e, 11-II-d, 11-IV-d, 12-IV-b, 12-V-b, 13-I-b, 13-II-b/c, 13-III-c,
Elongate entire and Spheriod hollow	Originating from palisade tissues and epidermal cells, respectively, often found to be articulated	*Acer oliverianum*, *Rhus chinensis*, *Betula delavayi*, *Corylus heterophylla*, *Sambucus adnata*, *Euptelea pleiosperma*, *Quercus acutissima*, *Pterocarya hupehensis*, *Ficus tikoua*, *Pittosporum truncatum*, *Cerasus maximowiczii*, *Tilia mandshurica*	Silicified cell class	Figure 1-14 Supplementary Figure 2-III-d, 3-II-b, 4-II-b, 4-III-f, 5-I-f, 8-I-d, 8-III-c, 9-II-c, 10-IV-b, 11-II-h, 11-III-b, 13-IV-b
Irregular articulated granulate	This type of phytolith was found in the fruit husk of *Aleurites moluccana*, has a twisted elongate morphology, can be highly variable, the surface has a granulate texture, found articulated forming a layer (single disarticulated phytoliths of this type could not be observed without breaking the layer)	*Aleurites moluccana*,	Silicified cell class	Figure 1-15 Supplementary Figure 7-II-g/h
Acute bulbosus	Originating from a fully silicified hair cell, has one ballooned end	*Corylus heterophylla*, *Corylus mandshurica*, *Sambucus adnata*, *Morus australis*, *Rosa helenae*	Hair tissue class	Figure 2-1 Supplementary Figure 4-III-d, 4-IV-e, 5-I-d, 11-I-b, 11-V-b
Acute uncinate	Originating from a not fully silicified hair cell, the tip is bent over to form a hook shape	*Smilax* sp., *Morus australis*	Hair tissue class	Figure 2-2 Supplementary Figure 10-II-d, 11-I-d
Acute	Originating from a not fully silicified hair cell, has a pointed shape, narrowing to a sharp apex and often slightly bent	*Aleurites moluccana*, *Leptopus chinensis*, *Lespedeza bicolor*, *Lespedeza cuneata*, *Smilax* sp., *Ficus tikoua*, *Morus australis*, *Pittosporum truncatum*, *Phellodendron amurense*	Hair tissue class	Figure 2-3 Supplementary Figure 7-II-d, 7-IV-b, 9-IV-e, 10-I-e, 10-II-f, 10-IV-c/d/f, 11-I-a, 11-II-g, 1-IV-c
Acute acicular	Originating from a not fully silicified hair cell, has the shape of a lance, sometimes a line could be observed along the axis of symmetry (it might be caused by the insufficient silicification)	*Morus australis*, *Sorbus multijuga*	Hair tissue class	Figure 2-4 Supplementary Figure 11-I-c, 12-II-d
Acute echinate	Originating from a not fully silicified hair cell, has many small spiny projections on the surface	*Ficus tikoua*,	Hair tissue class	Figure 2-5 Supplementary Figure 10-IV-e
Hair base	Originating from silicified hair base cells, has the shape of a floral hoop	*Acer komarovii*, *Acer tataricum* sub *ginnala, Acer ukurunduense*, *Corylus heterophylla*, *Corylus mandshurica*, *Sambucus adnata*, *Viburnum foetidum* var. *ceanothoides*, *Aleurites moluccana*, *Quercus acutissima*, *Quercus mongolica*, *Aesculus chinensis*, *Lespedeza cuneata*, *Ficus tikoua*, *Morus australis*, *Rosa acicularis*, *Paulownia fargesii*	Hair tissue class	Figure 2-6 Supplementary Figure 1-II-b, 2-IV-c, 3-I-c, 4-III-d, 4-IV-e, 5-I-d/e, 5-II-c, 7-II-e, 8-III-d, 8-IV-d, 9-I-e, 10-I-d, 10-IV-c, 11-I-a, 11-IV-c, 13-III-b
Trichome spheroid plicate/cavate	Possibly originating from silicified trichome tissue, has a spheroid body with a wrinkled surface, and is hollow inside	*Corylus heterophylla*, *Corylus mandshurica*, *Cornus controversa*, *Euptelea pleiosperma*, *Aesculus chinensis*, *Cerasus maximowiczii*, *Populus lasiocarpa*	Hair tissue class	Figure 2-7 Supplementary Figure 4-III-g, 4-IV-g, 5-IV-f, 8-I-e, 9-I-f, 11-III-d, 12-V-d
Ellipsoidal nodulate	Unknown origin, possibly originating from a silicified sclereid, has a spheroid to ellipsoidal shape with many rounded nodules on the surface	*Populus* sp.	Silicified cell class	Figure 2-8 Supplementary Figure 12-VI-d
Trichome fusiform cavate	Unknown origin, possibly originating from silicified trichome tissue, has a fusiform shape with an opening on one side and is hollow inside	*Cornus controversa*, *Cornus hemsleyi*, *Cornus schindleri* sub *poliophylla*	Hair tissue class	Figure 2-9 Supplementary Figure 5-IV-d, 6-I-d, 6-II-e

Most phytoliths observed in this study were found in leaves, except for Elongate entire (Fig. 1-10) which were also observed in the vine of *Ficus tikoua*, the twig of *Pittosporum truncatum* and *Tilia mandshurica*, and Irregular articulated granulate (Fig. 1-15), which were only observed in the fruit husk of *Aleurites moluccana*. Because many phytolith types have the same anatomical origin, to simplify the further analysis, we further classify the phytoliths types into 4 categories or classes:

- the **stomata class**, phytoliths that were formed in the stomata in the leaves, which includes the Stomate stellate;
- the **hair tissue class**, phytoliths that were formed in the hair tissues in the leaves, which includes the Trichome irregular tubercule, Trichome bulbous irregular, Acute bulbosus, Acute uncinate, Acute, Acute acicular, Acute echinate, Hair base, Trichome spheroid plicate/cavate, Trichome fusiform cavate;
- the **tracheid/vascular tissue class**, phytoliths that were formed in the tracheid/vascular tissues in the leaves, which included the Elongate facetate, Tracheary annulate/facetate geniculate, Tracheary annulate/facetate claviform, Tracheary annulate, Tracheary helical;
- the **silicified cell class**, phytoliths that were formed in the cells of mesophyll or epidermis in leaves/branches/fruit, which includes the Elongate brachiate geniculate, Irregular sinuate, Polygonal tabular, Spheroid favose, Elongate entire, Spheriod hollow, Ellipsoidal nodulate.

**Figure 3:**
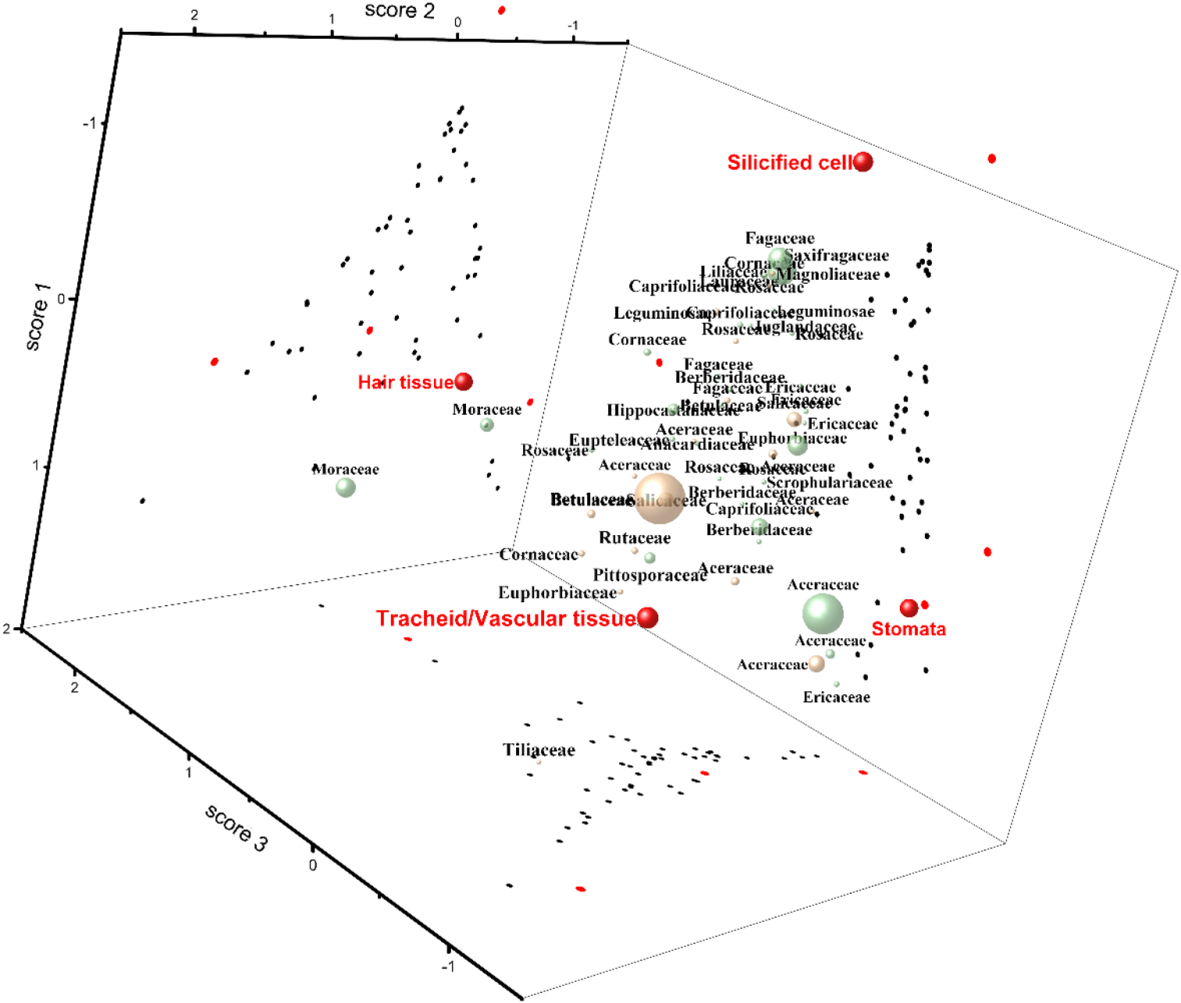
Relationship among specimens and phytoliths using PCA analysis. Red spheres: indicates the types of phytolith; Green spheres: represents specimens collected in southern China; Yellow spheres: represents specimens collected in northern China. The size of the Green and Yellow spheres relates to the total count of phytoliths of the specimen, the larger the sphere the more phytoliths identified in each specimen. The red and black dots are the projection of spheres on different quadrant. Refer to the result part for more details.

The total count of phytoliths in each specimen and the percentage of phytoliths in each category are reported in Table 3. We carried out a PCA analysis using this set of data, to find out the relationship between the phytoliths types and species. The result is reported in Fig. 3. We note that the spheres (the red spheres) that represent the four categories of phytolith types form a tetrahedron in the coordinate system Fig. 3, with each sphere occupying an apex of the tetrahedron, indicating that the four categories can be clearly separated. We further note that the spheres that represent the species are scattered throughout the coordinate system with their positions reflecting their relationship with the four phytolith type categories. This PCA closest relationship paradigm between phytolith type categories and the species suggests that phytoliths of the stomata class could be more representative of Aceraceae and Ericaceae, phytoliths of the hair tissue class could be more representative of Moraceae, phytoliths of the tracheid/vascular tissue class could be more representative of Tiliaceae and Euphorbiaceae, phytoliths of the silicified cell class could be more representative of Fagaceae, Saxifragaceae, Liliaceae, Magnoliaceae, Cornaceae, Rosaceae and Lauraceae.

**Table 3:** Phytolith percentage and phytolith count in studied specimens.

Family	Latin name	Stomata	Hair tissue	Tracheid/Vascular tissue	Silicified cell	Total count	Supplementary Figure
Aceraceae	*Acer caudatum* Wall	78.23	0.00	1.61	20.16	248	1-I
Aceraceae	*Acer komarovii* Pojark	25.56	34.59	2.26	37.59	133	1-II
Aceraceae	*Acer laxiflorum* Pax	68.96	0.00	2.45	28.58	1060	1-III
Aceraceae	*Acer mandshuricum* Maxim	33.65	0.00	30.77	35.58	208	2-I
Aceraceae	*Acer negundo* Linn	48.65	0.00	2.03	49.32	148	2-II
Aceraceae	*Acer oliverianum* Pax	33.60	0.00	4.23	62.17	497	2-III
Aceraceae	*Acer tataricum* sub *ginnala* (Maximowicz) Wesmael	79.19	3.17	1.36	16.29	442	2-IV
Aceraceae	*Acer ukurunduense* Trautv. et Mey	7.69	6.29	27.27	58.74	143	3-I
Anacardiaceae	*Rhus chinensis* Mill	1.83	0.00	36.70	61.47	109	3-II
Anacardiaceae	*Rhus potaninii* Maxim	0.00	0.00	23.53	76.47	34	3-III
Berbeidaceae	*Berberis poiretii* Schneid	0.00	0.00	50.00	50.00	2	NA
Berberidaceae	*Berberis diaphana* Maxin	24.32	0.00	25.23	50.45	111	3-IV
Berberidaceae	*Berberis dictyophylla* Franch	3.91	0.00	24.22	71.88	128	3-V
Berberidaceae	*Mahonia bealei* (Fort.) Carr	35.43	0.00	21.26	43.31	127	4-I
Betulaceae	*Betula delavayi* Franch	3.36	0.00	27.73	68.91	119	4-II
Betulaceae	*Corylus heterophylla* Fisch. ex Trautv	0.45	1.72	47.62	50.21	2667	4-III
Betulaceae	*Corylus mandshurica* Maxim	1.50	19.00	41.50	38.00	200	4-IV
Caprifoliaceae	*Sambucus adnata* Wall. ex DC	5.80	7.25	5.80	81.16	138	5-I
Caprifoliaceae	*Viburnum foetidum* Wall. var. *ceanothoides* (C. H. Wright) Hand.-Mazz	2.65	9.73	0.00	87.61	113	5-II
Caprifoliaceae	*Viburnum* sp.	33.41	0.00	20.47	46.12	425	5-III
Cornaceae	*Cornus controversa* Hemsley	11.25	25.63	36.25	26.88	160	5-IV
Cornaceae	*Cornus hemsleyi* C. K. Schneider & Wangerin	0.60	2.41	4.22	92.77	166	6-I
Cornaceae	*Cornus schindleri* sub *poliophylla* (C. K. Schneider & Wangerin) Q. Y. Xiang	2.19	26.23	5.46	66.12	183	6-II
Ericaceae	*Rhododendron calophytum* Franch	31.06	0.00	0.00	68.94	132	6-III
Ericaceae	*Rhododendron concinnum* Hemsl	32.43	0.00	0.90	66.67	111	6-IV
Ericaceae	*Rhododendron rubiginosum* Franch	25.42	0.00	0.85	73.73	118	7-I
Ericaceae	*Rhododendron strigillosum* Franch	85.62	0.00	0.65	13.73	153	NA
Euphorbiaceae	*Aleurites moluccana* (L.) Willd	34.04	2.84	0.71	62.41	141	7-II
Euphorbiaceae	*Flueggea suffruticosa* (Pall.) Baill	0.00	0.00	65.94	34.06	138	7-III
Euphorbiaceae	*Leptopus chinensis* (Bunge) Pojark	13.79	13.79	34.48	37.93	29	7-IV
Eupteleaceae	*Euptelea pleiosperma* J. D. Hooker & Thomson?	0.00	5.47	35.16	59.38	128	8-I
Fagaceae	*Fagus engleriana* Seem	0.18	0.00	2.65	97.17	566	8-II
Fagaceae	*Quercus acutissima* Carr	6.04	6.71	16.11	71.14	149	8-III
Fagaceae	*Quercus mongolica* Fischer ex Ledebour	4.70	0.00	25.50	69.80	149	8-IV
Hippocastanaceae	*Aesculus chinensis* Bunge	9.69	17.99	13.15	59.17	289	9-I
Juglandaceae	*Pterocarya hupehensis* Skan	0.88	0.00	15.04	84.07	113	9-II
Lauraceae	*Machilus microcarpa* Hemsl	4.12	0.00	2.58	93.30	194	9-III
Leguminosae	*Lespedeza bicolor* Turcz	3.50	13.50	2.50	80.50	200	9-IV
Leguminosae	*Lespedeza cuneata* (Dumont de Courset) G. Don	10.16	3.91	0.00	85.94	128	10-I
Liliaceae	*Smilax* sp.	3.58	1.00	0.72	94.71	699	10-II
Magnoliaceae	*Oyama sieboldii* (K. Koch) N. H. Xia & C. Y. Wu	0.00	0.00	5.67	94.33	141	10-III
Moraceae	*Ficus tikoua* Bur	0.84	62.29	3.91	32.96	358	10-IV
Moraceae	*Morus australis* Poir	0.00	97.38	1.31	1.31	534	11-I
Pittosporaceae	*Pittosporum truncatum* Pritz	5.22	1.12	54.10	39.55	268	11-II
Rosaceae	*Cerasus maximowiczii* (Rupr.) Kom	31.08	3.60	6.76	58.56	222	11-III
Rosaceae	*Rosa acicularis* Lindl	2.94	3.68	13.97	79.41	136	11-IV
Rosaceae	*Rosa helenae* Rehder & E. H. Wilson	5.88	32.03	18.30	43.79	153	11-V
Rosaceae	*Sorbaria sorbifolia* (Linn.) A. Br	0.90	0.00	7.21	91.89	111	12-I
Rosaceae	*Sorbus multijuga* Koehne	15.83	0.83	0.00	83.33	120	12-II
Rosaceae	*Spiraea longigemmis* Maxim	13.56	0.00	31.36	55.08	118	12-III
Rutaceae	*Phellodendron amurense* Rupr	0.00	1.32	57.89	40.79	152	12-IV
Salicaceae	*Populus lasiocarpa* Oliv	31.13	2.11	0.53	66.23	379	12-V
Salicaceae	*Populus* sp.	0.59	0.00	48.52	50.89	338	12-VI
Salicaceae	*Salix ernestii* C. K. Schneid	34.09	0.00	6.82	59.09	44	13-I
Saxifragaceae	*Philadelphus schrenkii* Rupr	0.00	0.00	1.87	98.13	107	13-II
Scrophulariaceae	*Paulownia fargesii* Franch	37.04	7.41	4.44	51.11	135	13-III
Tiliaceae	*Tilia mandshurica* Rupr. et Maxim	0.00	0.00	96.08	3.92	102	13-IV
Ulmaceae	*Zelkova schneideriana* Hand.-Mazz	0.00	0.00	0.00	100.00	3	NA

NA indicated that the photograph of this specimen was not provided in the supplementary file. *Berberis poiretii* produced Acute and Tracheid annulate; *Rhododendron strigillosum* produced the same types of phytoliths as those from genus *Rhododendron*; *Zelkova schneideriana* only produced Acute.

The % indicated that the numbers of the column refer to the percentage of this category, and the total count indicate the number of phytoliths counted in the 100 fields of view under 400 × microscope.

### Phytolith production in the studied species

To evaluate phytolith production in each specimen, we adapted the production index (PI) used by Pearce and Ball (2019)^15^:

- NP (non producer): no phytoliths observed
- R (rare): one or two phytoliths observed
- U (uncommon): 3–30 phytoliths observed
- C (common): 30–100 phytoliths observed
- A (abundant): more than 100 phytoliths observed

Of 110 species we analyzed, 58 produced phytoliths and 52 were non phytolith producers Table 1. The production index for 58 phytolith producers was mostly recognized as abundant (A) and common (C), except for *Berberis poiretii* (which was rare), and *Leptopus chinensis* and *Zelkova schneideriana* (which are uncommon).

Among the phytolith producers, 21 species were collected from Northern China (Changbai Mountain and Beijing) and 37 were from Southern China (Gongga Mountain). To compare phytolith production between the two regions, we applied an independent-samples Mann–Whitney U test using the data in Table 3. The results showed that phytolith production in the stomata class (Sig. = 0.147), the hair tissue class (Sig. = 0.792) and the silicified cell class (Sig. = 0.226) showed no significant differences between the two regions, however, phytolith production in the tracheid/vascular tissue class (Sig. = 0.028) was significantly different between Northern and Southern China. Also, despite some differences in the taxa, the total count of phytolith showed no significant differences between the two regions (Sig. = 0.601). Such results indicated that although the tracheid/vascular tissue class differed between the two regions, the production of most other phytoliths types might not be influenced by regional differences. The differences in the production of the tracheid/vascular tissue class might reflect the different hydrothermal conditions in the two regions.

## Discussion and conclusions

It is widely known that in general, woody plants produce fewer phytoliths than grasses^4,5^. The results of our study are consistent with the previous studies. Only 58 out of the 110 species we analyzed were phytoliths producers. Most of the phytoliths we observed were extracted from leaves, the other plant parts, such as twigs and fruits typically showing a lack of silicification. Phytolith types belonging to the silicified cell class make up the largest portion of the phytoliths produced by the 58 phytolith producing taxa, followed by the stomata class, the tracheid/vascular class and the hair tissue class. Species belonging to the same genus usually produced the same types of phytoliths, and the phytolith production was typically similar. However, we found that phytolith types and production in species belonging to different genera of the same family can be very different. Such results suggest the possibility of identification of taxa on the genus level using phytolith analysis, which is in consist with the study of grasses^22^, however, studies that involve more species and more samples of species are needed to confirm such findings.

To date, no especially diagnostic types of phytoliths have been identified for broad-leaved trees in general or a certain family. After reviewing other phytolith studies of species belonging to the broad-leaved trees^12,15,46–52,55,56^ (also see Table 4, we here propose several phytolith types that have the potential to be diagnostic to broad-leaved trees: Elongate brachiate geniculate (Fig. 1-3), Polygonal tabular (Fig. 1-5), Elongate facetate (Fig. 1-8), Tracheary annulate/facetate geniculate (Fig. 1-9) and Tracheary annulate/facetate claviform (Fig. 1-10). Because these types of phytoliths are rarely seen in grasses and have been extracted from broad-leaved tree taxa in other studies, we suggest that they might have the potential to be diagnostic types for broad-leaved trees. Although some types of phytoliths have distinct morphological differences with other types (such as Trichome irregular tubercule (Fig. 1-6), Trichome spheroid plicate/cavate (Fig. 2-7), Ellipsoidal nodulate (Fig. 2-8) and Trichome fusiform cavate (Fig. 2-9), considering the lack of cross-examination of these types, further studies were needed to evaluate their potential in being diagnostic types. The Acute acicular (Fig. 2-4) and Acute echinate (Fig. 2-5) were only observed in Moraceae plants^4,7,48^, combined with our results, they might be the potential diagnostic types for Moraceae, while observation of more specimens from Moraceae and other plants was needed to confirm this finding. Although Irregular sinuate phytoliths were observed in many broad-leaved trees, they were also observed in many ferns^4,45,54,65^, thus they were not proposed as the potential diagnostic types for broad-leaved trees. The Irregular articulated granulate (Fig. 1-15) which we found in the fruit husk of *Aleurites moluccana* (which could be used as food or sauce in Malaysia and Indonesia), is also noteworthy as it has not been reported yet. Such silicification in fruit husks might be a protection strategy^22,66^, and the presence of this type may provide insight into ancient plant resource exploitation.

**Table 4:** Comparison of phytoliths nomenclature and evaluation of their potential in being diagnostic types for broad-leaved trees.

Current name	Former names	Potential of being diagnostic types for broad-leaved trees
Stomate stellate (Fig. 1-1 and 1-2)	Silicified stomata^4,46,52^, stomata phytolith^45^, stomata^48^, stomata dicotyledon type^51^, stomata cell^12^, stomata hairy/special^56^, stoma^50^	This type of phytoliths have been commonly observed in plants, however, the silicified stomata with radiative/stellate margins might of some potential in being the diagnostic type for broad-leaved trees
Elongate brachiate geniculate (Fig. 1-3)	Silicified sclereid^7^, Y-shaped^4^, sclereid phytolith^45^, brachiates^50^	This type of phytoliths have been frequently reported to be observed in broad-leaved trees, thus it might be of high potential to be a diagnostic type for broad-leaved trees
Irregular sinuate (Fig. 1-4)	Silicified epidermal cell^46^, anticlinal epidermal phytolith^7^, anticlinal^4,45^, anticlinal epidermal cell^48^, silicified tissue of the leaf epidermis composed of puzzle-piece-shaped cells^52^, jigsaw epidermal cell^50,51^, broad-leaf-types^9^; jigsaw-shaped epidermal phytolith^5^; epidermal jig-saw^12^, anticlinal epidermal cell^49^, stellate^55^, tabular sinuate^56^, irregular psilate sinuate^15^	This type of phytoliths have been frequently reported to be observed in broad-leaved trees, however, they have also been reported to be observed in ferns, thus it might of low potential in being a diagnostic type for broad-leaved trees
Polygonal tabula (Fig. 1-5)	Silicified epidermal cell^46^, polyhedral epidermal phytolith^7^, polygonal^4,45, 58^; epidermal polygonal^12^, polyhedral epidermal cell^49^, tabular irregular^56^, isodiametric epidermal cell^50^, polygonal psilate entire^15^	This type of phytoliths have been frequently reported to be observed in broad-leaved trees and have distinct difference with those from grasses (mostly rectangle-shaped), thus it might of high potential in being a diagnostic type for broad-leaved trees
Trichome irregular tubercule (Fig. 1-6)	First reported in this study	This type of phytoliths belong to the hair/trichome class, however, it has distinct morphology that differs from others, and have not been observed in grasses, thus it might have the potential in being a diagnostic type for broad-leaved trees
Trichome bulbous irregular (Fig. 1-7)	Polygonal^4,45^	This type of phytoliths have not been reported in grasses and it has distinct morphology that differs from others; thus, it might have the potential in being a diagnostic type for broad-leaved trees
Elongate facetate (Fig. 1-8)	Elongate multifaceted^7^, tracheid phytolith^45^, elongate body with a faceted surface^52^, broad-leaf-types^4,9^; facetate terminal tracheid phytolith^5^	This type of phytoliths have been frequently reported to be observed in broad-leaved trees, thus it might of high potential in being a diagnostic type for broad-leaved trees
Tracheary annulate/facetate geniculate (Fig. 1-9)	Elongate multifaceted^7^, tracheid phytolith^45^, multifaceted polyhedral^48^, elongate body with a faceted surface^52^, broad-leaf-types^4^^,9^; facetate terminal tracheid phytolith^5^	This type of phytoliths have been frequently reported to be observed in broad-leaved trees, thus it might of high potential in being a diagnostic type for broad-leaved trees
Tracheary annulate/facetate claviform (Fig. 1-10)	Elliptical multifaceted phytolith^7^, tracheid phytolith^45^, multifaceted polyhedral^48^, broad-leaf-types^4,9^; facetate terminal tracheid phytolith^5^	This type of phytoliths have been frequently reported to be observed in broad-leaved trees, thus it might of high potential in being a diagnostic type for broad-leaved trees
Tracheary annulate (Fig. 1-11)	Tracheary elements^46^, tracheid phytolith^7,49^, cylindric^4^, tracheid phytolith^45^, rod with a ring- or spiral-shaped surface derived from tracheid^52^, vessels^51^, simple tracheid phytolith^5^; vessel member^12^, spiracle tracheid^56^, tracheary annulate^15,50^	This type of phytoliths have been commonly observed in plants, thus it might have low potential in being a diagnostic type for broad-leaved trees
Tracheary helical (Fig. 1-2)	Tracheary elements^46^, tracheid phytolith^7,49^, cylindric (Wang and Lu^4^, tracheid phytolith^45^, rod with a ring- or spiral-shaped surface derived from tracheid^52^, vessels with spiral thickening^51^, simple tracheid phytolith^5^; cylindric spiraling^55^, spiracle tracheid^56^, tracheary helical^15,50^	This type of phytoliths have been commonly observed in plants, thus it might have low potential in being a diagnostic type for broad-leaved trees
Spheroid favose (Fig. 1-13)	Silicified end walls of palisade mesophyll cells^46,49^, mesophyll phytolith^7,45^, favose^4^, mesophyll cells^51^, favose phytolith^56^, silicified mesophyll^50^, circular/ovate^15^	This type of phytoliths have been commonly observed in plants, thus it might have low potential in being a diagnostic type for broad-leaved trees
Elongate entire and Spheriod hollow (Fig. 1-14)	Silicified palisade mesophyll cell walls^46^, silicified palisade^4^, palisade phytolith^56^	This type of phytoliths have been commonly observed in plants, thus it might have low potential in being a diagnostic type for broad-leaved trees
Irregular articulated granulate (Fig. 1-15)	First reported in this study	This type of phytoliths have only been observed in the fruit husk of *Aleurites moluccana*, thus it might be of high potential in being a diagnostic type for this plant
Acute bulbosus (Fig. 2-1)	Long point^4,9^; hair^12^, lanceolate^56^, acute bulbosis^15^	This type of phytoliths have been commonly observed in plants, thus it might have low potential in being a diagnostic type for broad-leaved trees; however, the Acute type of phytoliths in woody plants were commonly larger than in Poaceae plants, the morphometric approach might help to increase the potential of Acute type of phytoliths in being a diagnostic type for broad-leaved trees
Acute uncinate (Fig. 2-2)	Silicified epidermal hair^46^, thin, curved hair cell phytoliths^7^, long point^4,9^	This type of phytoliths have been commonly observed in plants, thus it might have low potential in being a diagnostic type for broad-leaved trees; however, the Acute type of phytoliths in woody plants were commonly larger than in Poaceae plants, the morphometric approach might help to increase the potential of Acute type of phytoliths in being a diagnostic type for broad-leaved trees
Acute (Fig. 2-3)	Silicified epidermal hair^46^, long point^4,9^; long, threadlike nonsegmented hair phytolith^7^, square proximal hair cell^48^, trichomas^51^, hair^12,56^, acicular psilate unsegmented hair^49^, arcicular hair cell^55^, arcicular^50^, acute^15^	This type of phytoliths have been commonly observed in plants, thus it might have low potential in being a diagnostic type for broad-leaved trees; however, the Acute type of phytoliths in woody plants were commonly larger than in Poaceae plants, the morphometric approach might help to increase the potential of Acute type of phytoliths in being a diagnostic type for broad-leaved trees
Acute acicular (Fig. 2-4)	Nonsegmented hair phytolith^7^, long point^4,9^	This type of phytoliths have been reported only being observed in Moraceae plants, thus it might have high potential in being a diagnostic type for Moraceae
Acute echinate (Fig. 2-5)	Hair phytolith with small spines^7^, armed hair^48^, long point^4,9^; hair^12^, long acicular granulate segmented hair^49^	This type of phytoliths have been reported only being observed in Moraceae plants, thus it might have high potential in being a diagnostic type for Moraceae; however a confuser from some grasses (typically Asteraceae) showed similar morphology, but the confusers were observed to be segmented, while in Moraceae they were all nonsegmented
Hair base (Fig. 2-6)	Silicified epidermal hair base^46^, hair base phytolith^7,48^, silicified hair base^4^, hair base^12,49,55,56^	This type of phytoliths have been commonly observed in plants, thus it might have low potential in being a diagnostic type for broad-leaved trees
Trichome spheroid plicate/cavate (Fig. 2-7)	Decorated sphere^48^, hair base^12^, ovate striate^55^	This type of phytoliths have been reported to be observed in some broad-leaved trees and have not been reported to be observed in grasses, thus it might have the potential in being a diagnostic type for broad-leaved trees; however, this type of phytoliths seemed to be thin-walled and might hardly be preserved in sediments
Ellipsoidal nodulate (Fig. 2-8)	Spherical nodular^45^	This type of phytoliths have been rarely reported, unlike the common spherical phytolith observed in Palmaceae, this type of phytoliths were larger (over 20 microns in diameter) and mostly not spherical but ellipsoidal, thus it might have the potential in being a diagnostic type for broad-leaved trees or genera *Populus*
Trichome fusiform cavate (Fig. 2-9)	First reported in this study	This type of phytoliths belong to the hair/trichome class, and it has distinct morphology that differs from others, and it has not been observed in grasses, thus it might have the potential in being a diagnostic type for broad-leaved trees; however, this type of phytoliths seemed to be thin-walled and might hardly be preserved in sediments

In this study, we have provided an illustration of several distinct phytolith types we observed in the common broad-leaved trees in temperate China, and reported that there appears to be little difference in broad-leaved trees phytolith production between the northern and the southern regions. Although we have proposed several specific phytoliths types as potentially diagnostic (which we believe to be reliable), pending further confirming research involving more taxa and samples, researchers should not solely use our findings as identification criteria, but rather as a guidance and reference for the future studies.

## Supplementary information

Supplementary Information.

## Data Availability

The raw materials of studied species and slides of phytoliths involved this study can be found in the phytolith lab at the Institute of Geology and Geophysics, Chinese Academy of Sciences.
