# Supplementary material for: Phytoliths in selected broad-leaved trees in China
Source: Sci Rep. 2020 Sep 23;10:15577. doi: 10.1038/s41598-020-72547-w (PMC7512002; doi:10.1038/s41598-020-72547-w)

Supplementary Figure 1. Phytoliths types that observed in *Acer caudatum*, *Acer komarovii*, and *Acer laxiflorum*.

Supplementary Figure 2. Phytoliths types that observed in *Acer mandshuricum*, *Acer negundo*, *Acer oliverianum*, and *Acer tataricum* sub *ginnala*.

Supplementary Figure 3. Phytoliths types that observed in *Acer ukurunduense*, *Rhus chinensis*, *Rhus potaninii*, *Berberis diaphana*, and *Berberis dictyophylla*.

Supplementary Figure 4. Phytoliths types that observed in *Mahonia bealei*, *Betula delavayi*, *Corylus heterophylla*, and *Corylus mandshurica*.

Supplementary Figure 5. Phytoliths types that observed in *Sambucus adnata*, *Viburnum foetidum* var. *ceanothoides*, *Viburnum* sp., and *Cornus controversa*.

Supplementary Figure 6. Phytoliths types that observed in *Cornus hemsleyi*, *Cornus schindleri* sub *poliophylla*, *Rhododendron calophytum*, and *Rhododendron concinnum*.

Supplementary Figure 7. Phytoliths types that observed in *Rhododendron rubiginosum*, *Aleurites moluccana*, *Flueggea suffruticosa*, and *Leptopus chinensis.*

Supplementary Figure 8. Phytoliths types that observed in *Euptelea pleiosperma*, *Fagus engleriana*, *Quercus acutissima*, and *Quercus mongolica*.

Supplementary Figure 9. Phytoliths types that observed in *Aesculus chinensis*, *Pterocarya hupehensis*, *Machilus microcarpa*, and *Lespedeza bicolor*.

Supplementary Figure 10. Phytoliths types that observed in *Lespedeza cuneata*, *Smilax* sp., *Oyama sieboldii*, and *Ficus tikoua*.

Supplementary Figure 11. Phytoliths types that observed in *Morus australis*, *Pittosporum truncatum*, *Cerasus maximowiczii*, *Rosa acicularis*, and *Rosa helenae*.

Supplementary Figure 12. Phytoliths types that observed in *Sorbaria sorbifolia*, *Sorbus multijuga*, *Spiraea longigemmis*, *Phellodendron amurense*, *Populus lasiocarpa*, and *Populus* sp..

Supplementary Figure 13. Phytoliths types that observed in *Salix ernestii*, *Philadelphus schrenkii*, *Paulownia fargesii*, and *Tilia mandshurica*.

# Supplementary Figure 1.

I. *Acer caudatum*, a. Stomate stellate, b. not fully silicified Polygonal tabular, c. articulated Polygonal tabular, d. Spheroid favose, e. Tracheary annulate.

II. *Acer komarovii*, a. Stomate stellate, b. Hair base, c. Irregular sinuate, d. Spheroid favose, e. Tracheary annulate.

III. *Acer laxiflorum*, a. Stomate stellate, b. not fully silicified Polygonal tabular, c. articulated Polygonal tabular, d. Spheroid favose, e. Tracheary annulate.


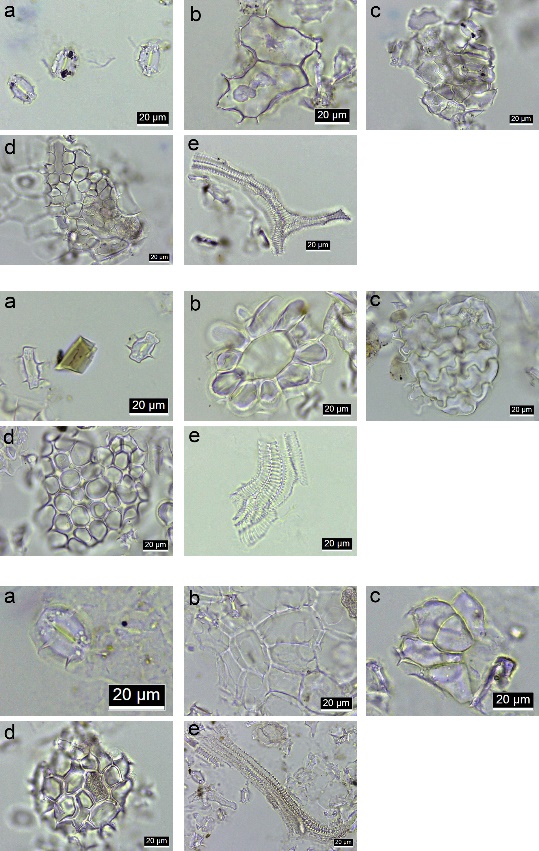


# Supplementary Figure 2.

I. *Acer mandshuricum*, a. Stomate stellate, b. not fully silicified Polygonal tabular, c. Polygonal tabular, d. Spheroid favose, e. Tracheary annulate.

II. *Acer negundo*, a. Stomate stellate, b. not fully silicified Polygonal tabular, c. Spheroid favose, d. Tracheary annulate.

III. *Acer oliverianum*, a. Stomate stellate, b. not fully silicified Polygonal tabular, c. Polygonal tabular, d. Elongate entire and Spheriod hollow, e. Spheroid favose, f. Tracheary annulate.

IV. *Acer tataricum* sub *ginnala*, a. Stomate stellate, b. Spheroid favose, c. Hair base, d. Polygonal tabular, e. Tracheary annulate.


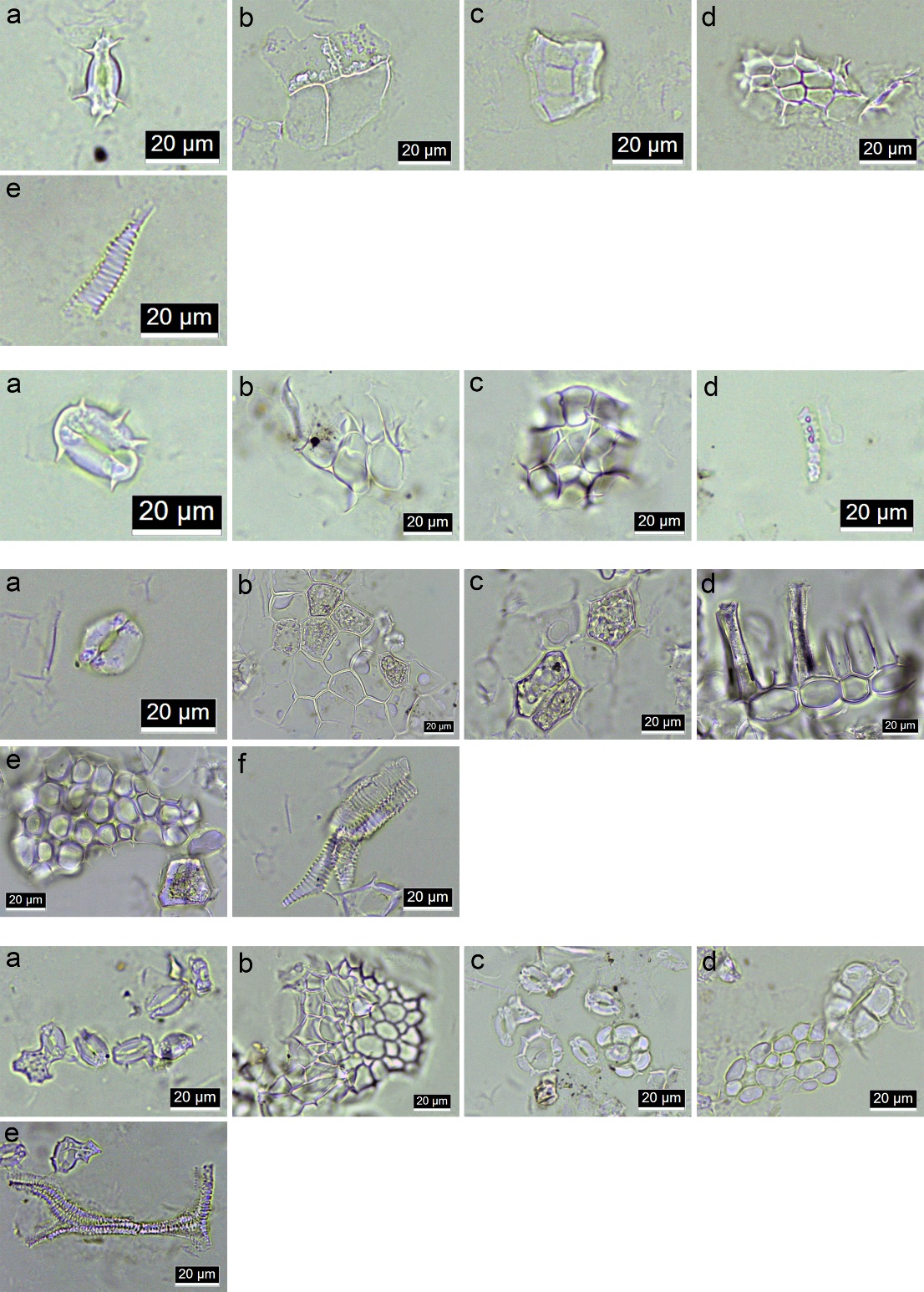


# Supplementary Figure 3.

I. *Acer ukurunduense*, a. Stomate stellate, b. Polygonal tabular, c. Hair base, d. Spheroid favose, e. articulated Tracheary annulate.

II. *Rhus chinensis*, a. Polygonal tabular, b. not fully silicified but articluated Elongate entire, c. articulated Spheroid favose and Tracheary annulate, d. Stomate stellate.

III. *Rhus potaninii*, a. not fully silicified Polygonal tabular, b. not fully silicified Polygonal tabular and Tracheary annulate, c. articulated Tracheary annulate.

IV. *Berberis diaphana*, a. Stomate stellate, b. Polygonal tabular, c. Tracheary annulate.

V. *Berberis dictyophylla*, a. Stomate stellate, b. Polygonal tabular, c. Tracheary annulate.


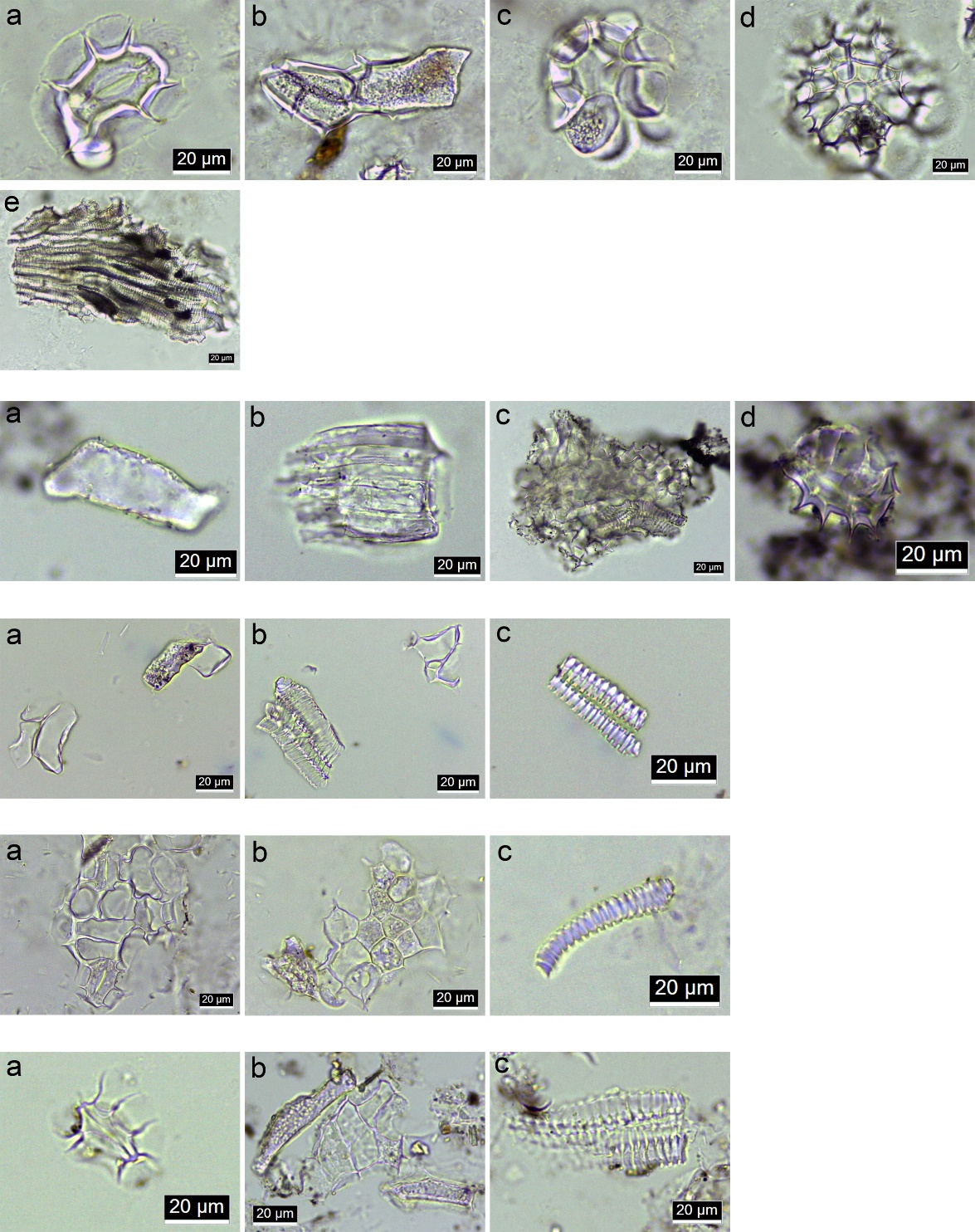


# Supplementary Figure 4.

I. *Mahonia bealei*, a. Stomate stellate, b. Polygonal tabular, c. Tracheary helical.

II. *Betula delavayi*, a. Stomate stellate, b. not fully silicified Elongate entire, c. articulated Polygonal tabular and Spheroid favose, d. Tracheary annulate, e. articulated Polygonal tabular.

III. *Corylus heterophylla*, a. Stomate stellate, b. articulated Irregular sinuate and Spheroid favose, c. Polygonal tabular, d. articulated Acute bulbosus and Hair base, e. Tracheary annulate, f. Elongate entire, g. Trichome spheroid plicate/cavate.

IV. *Corylus mandshurica*, a. Stomate stellate, b. articulated Irregular sinuate, c. articulated Spheroid favose and Polygonal tabular, d. Spheroid favose, e. articulated Acute bulbosus and Hair base, f. articulated Tracheary annulate, g. Tracheary annulate and Trichome spheroid plicate/cavate.


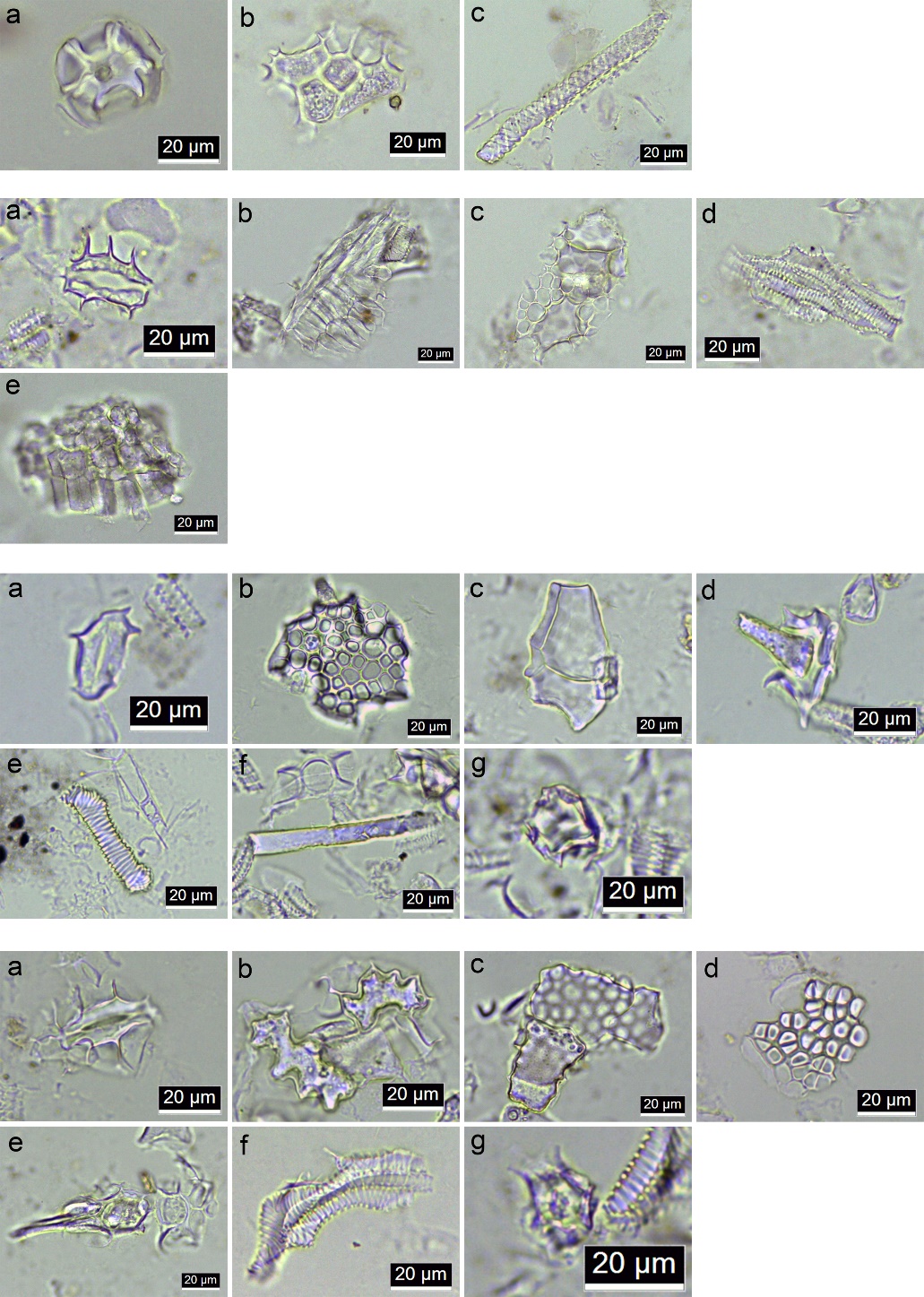


# Supplementary Figure 5.

I. *Sambucus adnata*, a. Stomate stellate, b. not fully silicified epidermis, c. articulated Polygonal tabular, d. articulated Acute bulbosus and Hair base, e. not fully silicified Hair base, f. articulated Spheriod hollow and Spheroid favose, g. Tracheary annulate.

II. *Viburnum foetidum* var. *ceanothoides*, a. Stomate stellate, b. Spheroid favose, c. Hair base.

III. *Viburnum* sp., a. Stomate stellate, b. not fully silicified Polygonal tabular, c. Polygonal tabular, d. Spheroid favose, e. Tracheary annulate.

IV. *Cornus controversa*, a. Stomate stellate, b. not fully silicified Polygonal tabular, c. Spheroid favose, d. Trichome fusiform cavate, e. Tracheary annulate, f. Trichome spheroid plicate/cavate.


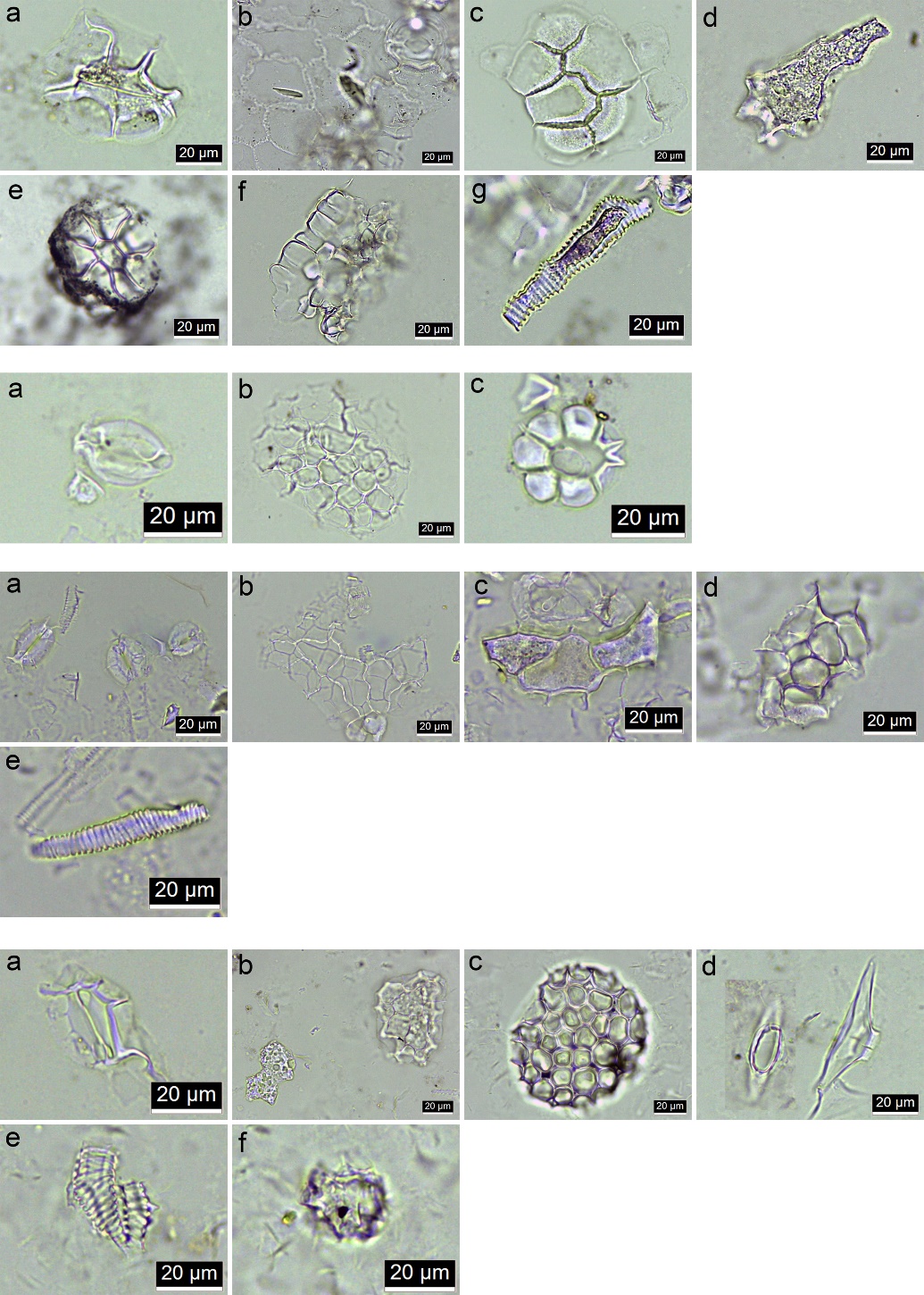


# Supplementary Figure 6.

I. *Cornus hemsleyi*, a. Stomate stellate, b. articulated Polygonal tabular, c. Spheroid favose, d. Trichome fusiform cavate, e. Tracheary annulate.

II. *Cornus schindleri* sub *poliophylla*, a. Stomate stellate, b. articulated Polygonal tabular, c. Spheroid favose, d. Trichome irregular tubercule, e. Trichome fusiform cavate, f. Trichome irregular tubercule, g. Tracheary annulate.

III. *Rhododendron calophytum*, a. Stomate stellate, b. Spheroid favose, c. Polygonal tabular, d. Elongate brachiate geniculate.

IV. *Rhododendron concinnum*, a. Stomate stellate, b. Spheroid favose, c. Polygonal tabular.


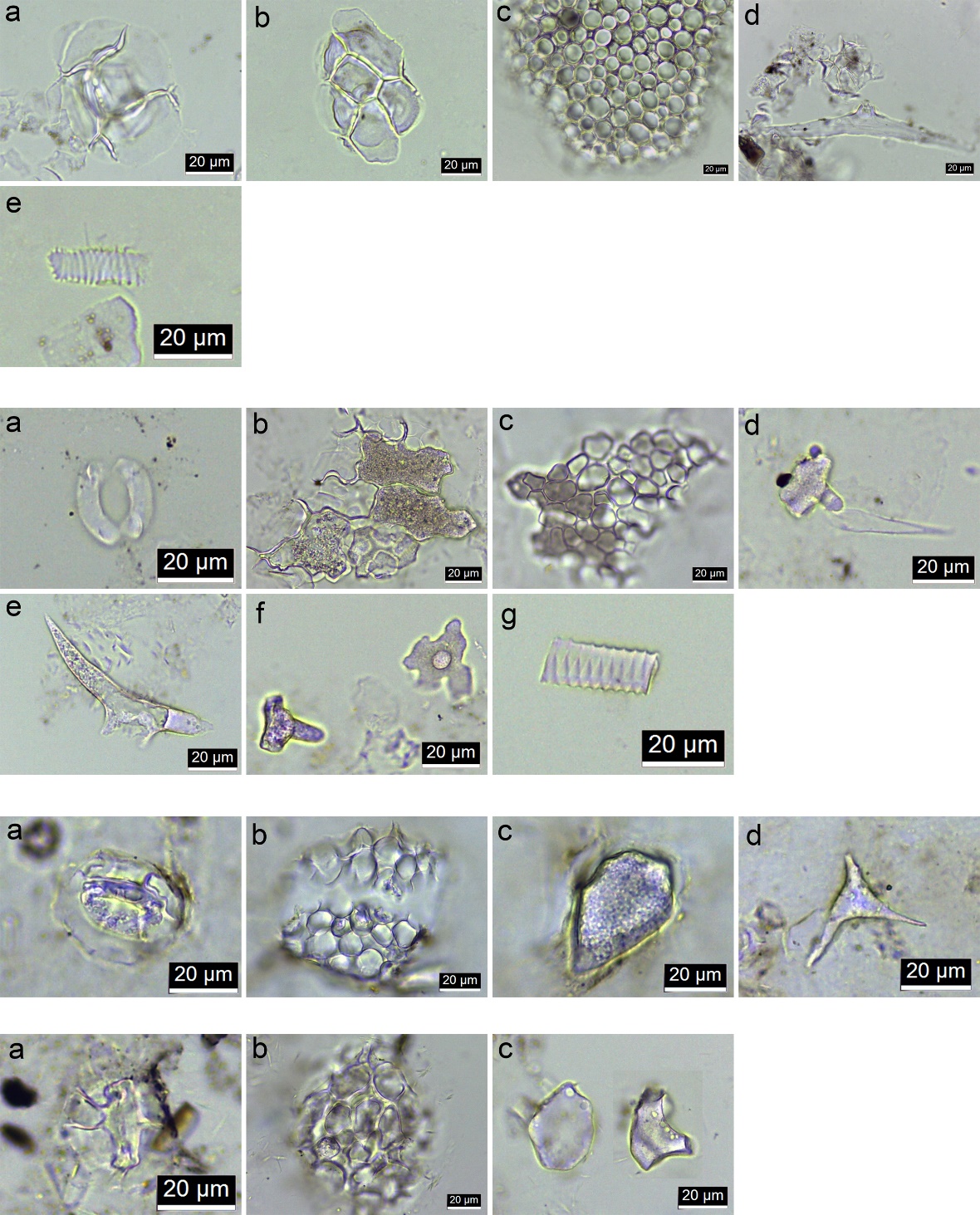


# Supplementary Figure 7.

I. *Rhododendron rubiginosum*, a. Stomate stellate, b. silicified epidermis, c. articulated Polygonal tabular, d. Spheroid favose.

II. *Aleurites moluccana*, a. Stomate stellate, b. not fully silicified Polygonal tabular, c. Spheroid favose, d. Acute, e. Hair base, f. Tracheary annulate, g. Irregular articulated granulate, h. single ones of Irregular articulated granulate.

III. *Flueggea suffruticosa*, a. Spheroid favose, b. articulated Polygonal tabular, c. Tracheary annulate.

IV. *Leptopus chinensis*, a. Stomate stellate, b. Acute, c. Tracheary annulate.


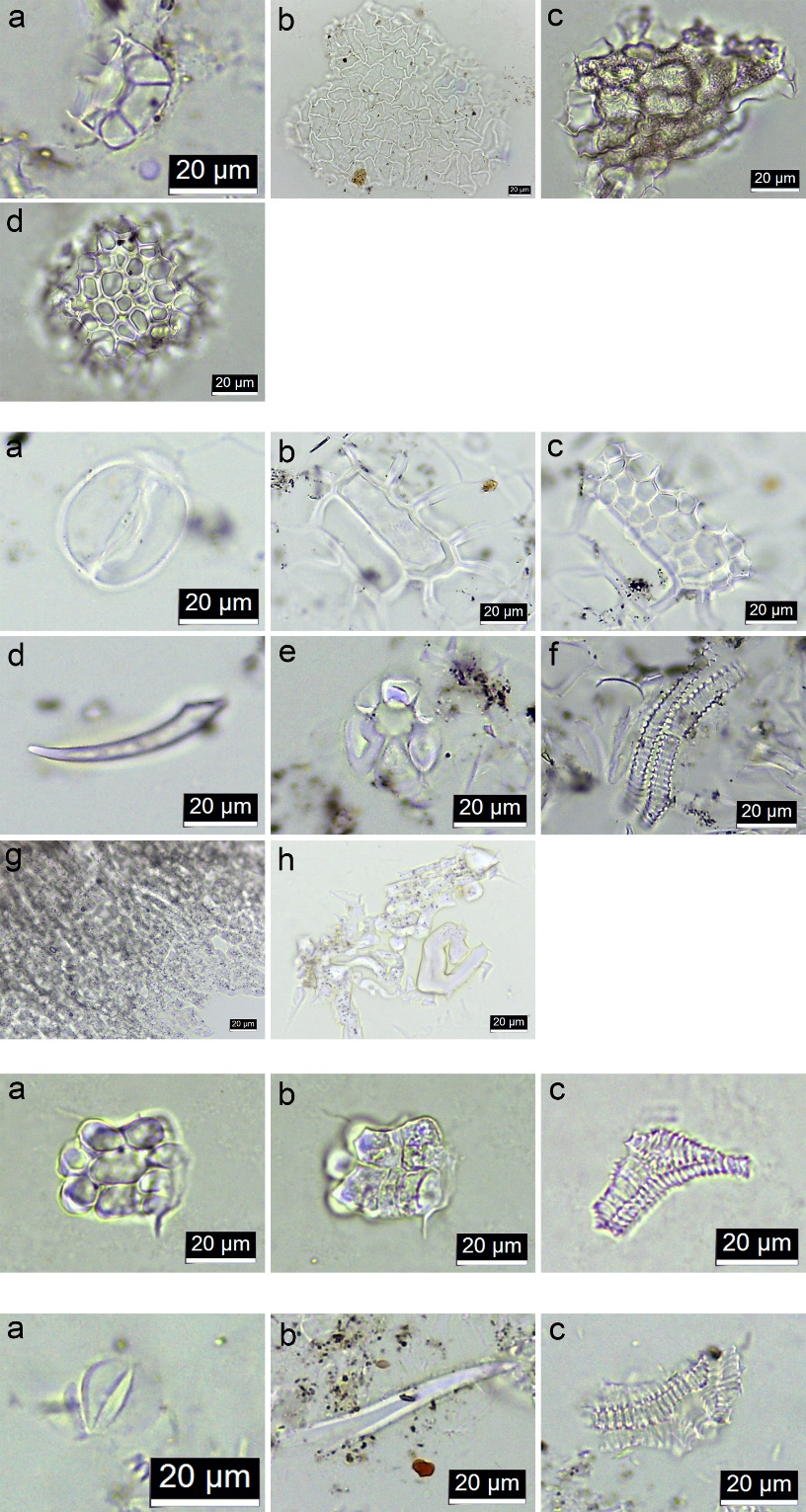


# Supplementary Figure 8.

I. *Euptelea pleiosperma*, a. articulated Polygonal tabular, b. Spheroid favose, c. Tracheary annulate, d. not fully silicified Elongate entire, e. Trichome spheroid plicate/cavate.

II. *Fagus engleriana*, a. Stomate stellate, b. articulated Irregular sinuate, c. articulated Spheroid favose, d. Tracheary annulate.

III. *Quercus acutissima*, a. Stomate stellate, b. articulated Polygonal tabular and Spheroid favose, c. not fully silicified Elongate entire, d. Hair base, e. Tracheary annulate.

IV. *Quercus mongolica*, a. Stomate stellate, b. articulated Polygonal tabular, c. Spheroid favose, d. Hair base, e. Tracheary annulate, f. Elongate brachiate geniculate.


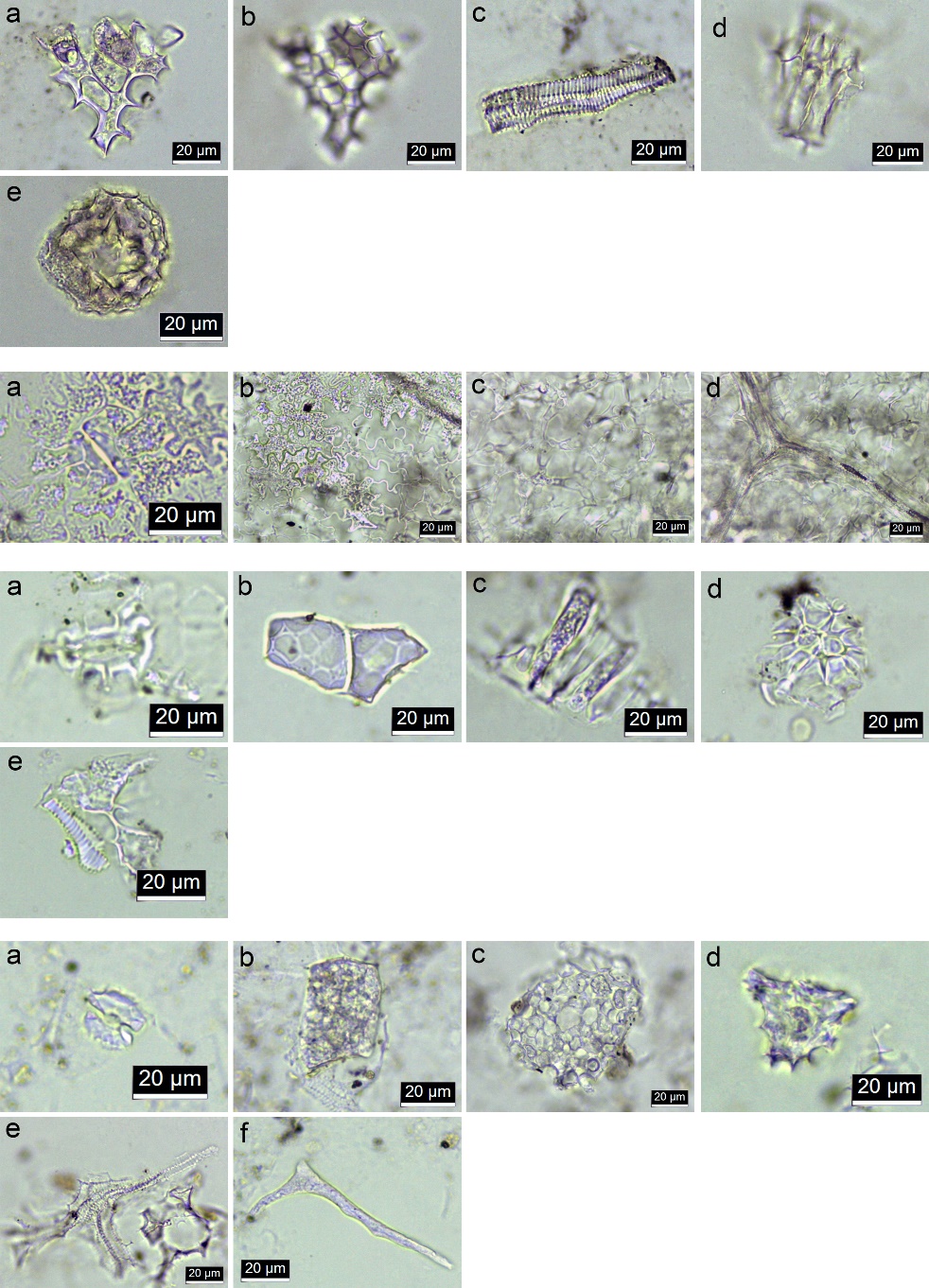


# Supplementary Figure 9.

I. *Aesculus chinensis*, a. Stomate stellate, b. silicified epidermis, c. Polygonal tabular, d. Spheroid favose, e. Hair base, f. Trichome spheroid plicate/cavate, g. articulated Tracheary annulate.

II. *Pterocarya hupehensis*, a. silicified epidermis, b. not fully silicified Irregular sinuate, c. not fully silicified Elongate entire, d. racheary annulate.

III. *Machilus microcarpa*, a. Elongate facetate, b. Tracheary facetate claviform, c. Elongate brachiate geniculate, d. Tracheary annulate.

IV. *Lespedeza bicolor*, a. Stomate stellate, b. articulated Irregular sinuate, c. articulated Irregular sinuate, d. Polygonal tabular, e. very large Acute, f. Tracheary helical.


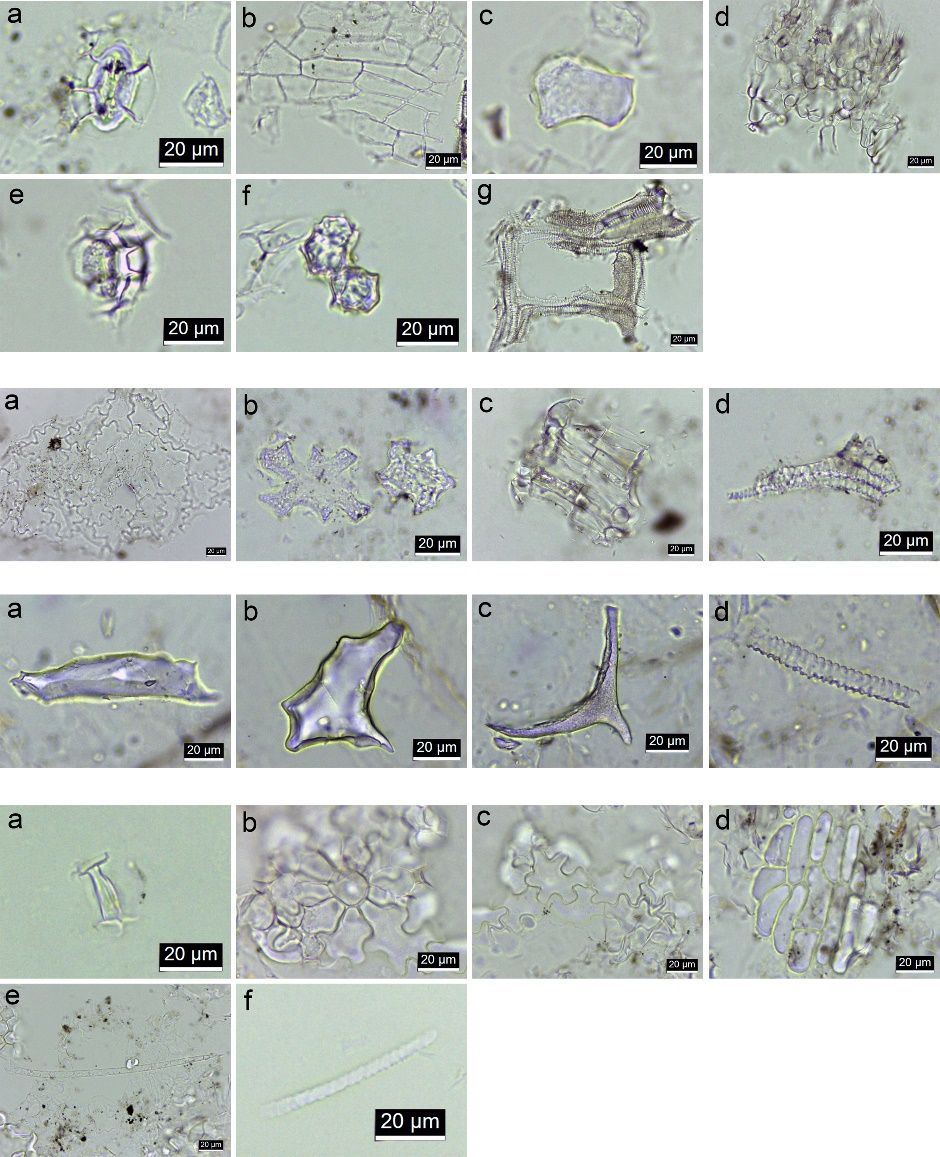


# Supplementary Figure 10.

I. *Lespedeza cuneata*, a. Stomate stellate, b. not fully silicified Polygonal tabular, c. Spheroid favose, d. Hair base, e. Acute.

II. *Smilax* sp., a. Stomate stellate, b. Polygonal tabular, c. Trichome bulbous irregular, d. Acute uncinate, e. Spheroid favose, f. Acute, g. articulated Tracheary annulate.

III. *Oyama sieboldii*, a. Stomate stellate, b. Tracheary annulate/facetate claviform, c. Tracheary annulate.

IV. *Ficus tikoua*, a. Polygonal tabular, b. Elongate entire extracted from the vine, c. Hair base surrounding an Acute, d. not fully silicified Acute, e. Acute echinate, f. Acute.


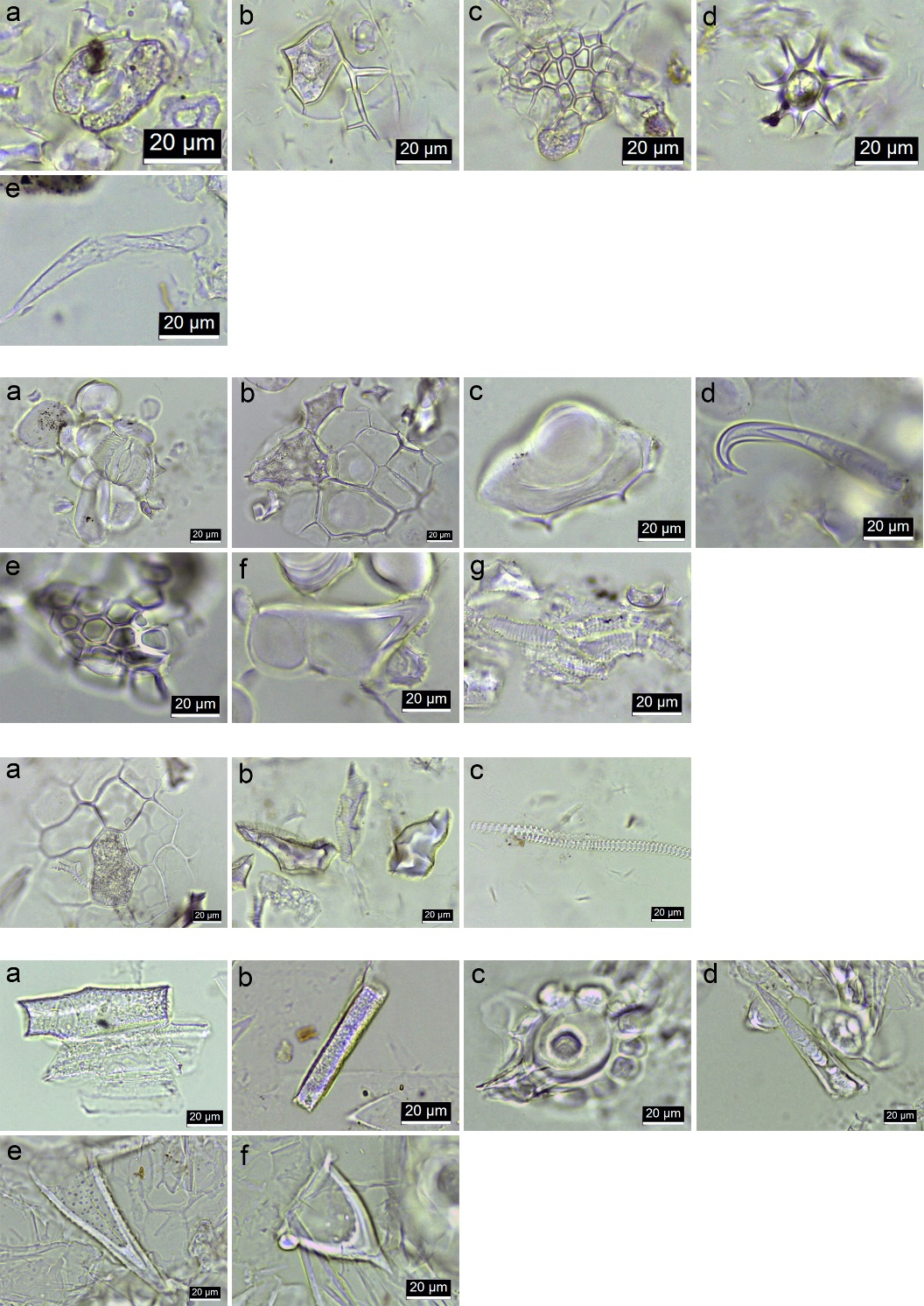


# Supplementary Figure 11.

I. *Morus australis*, a. Hair base surrounding an Acute, b. Acute bulbosus, c. Acute acicular, d. Acute uncinate.

II. *Pittosporum truncatum*, a. Stomate stellate, b. silicified epidermis, c. Irregular sinuate, d. Spheroid favose, e. Tracheary annulate/facetate geniculate, f. Elongate facetate, g. Acute, h. Elongate entire extracted from twig.

III. *Cerasus maximowiczii*, a. Stomate stellate, b. not fully silicified Elongate entire and Polygonal tabular, c. Tracheary annulate, d. Trichome spheroid plicate/cavate.

IV. *Rosa acicularis*, a. Stomate stellate, b. Polygonal tabular, c. Hair base, d. Spheroid favose.

V. *Rosa helenae*, a. Stomate stellate, b. Acute bulbosus, c. Tracheary annulate, d. not fully silicified Polygonal tabular.


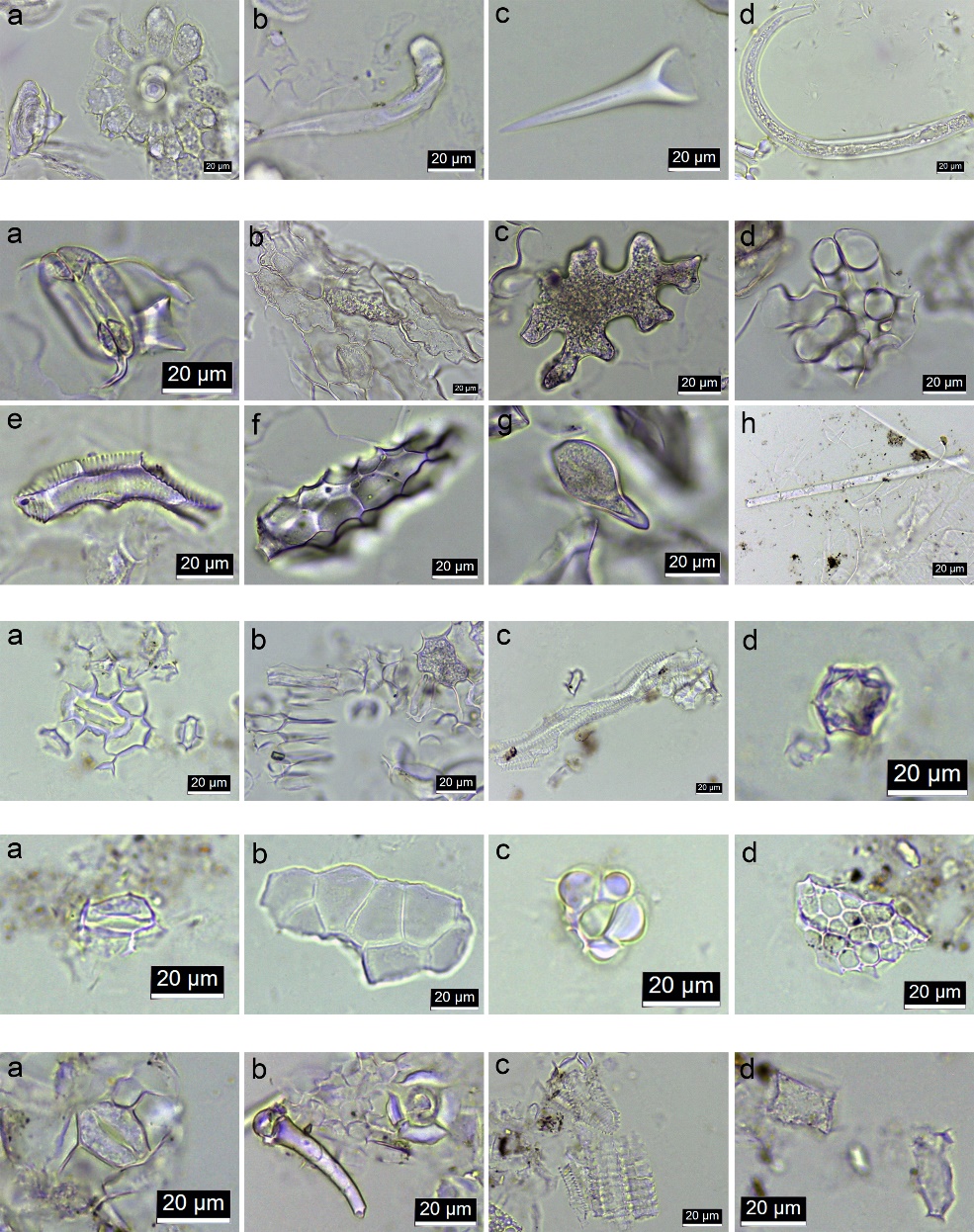


# Supplementary Figure 12.

I. *Sorbaria sorbifolia*, a. Stomate stellate, b. silicified epidermis, c. Tracheary annulate.

II. *Sorbus multijuga*, a. Stomate stellate, b. not fully silicified Polygonal tabular, c. Polygonal tabular, d. Acute.

III. *Spiraea longigemmis*, a. Stomate stellate, b. silicified epidermis, c. articulated Tracheary annulate.

IV. *Phellodendron amurense*, a. Irregular sinuate, b. Spheroid favose, c. Acute, d. Tracheary annulate.

V. *Populus lasiocarpa*, a. Stomate stellate, b. articulated Spheroid favose and Polygonal tabular, c. Tracheary annulate, d. Trichome spheroid plicate/cavate.

VI. *Populus* sp., a. Stomate stellate, b. silicified epidermis, c. articulated Tracheary annulate, d. Ellipsoidal nodulate.


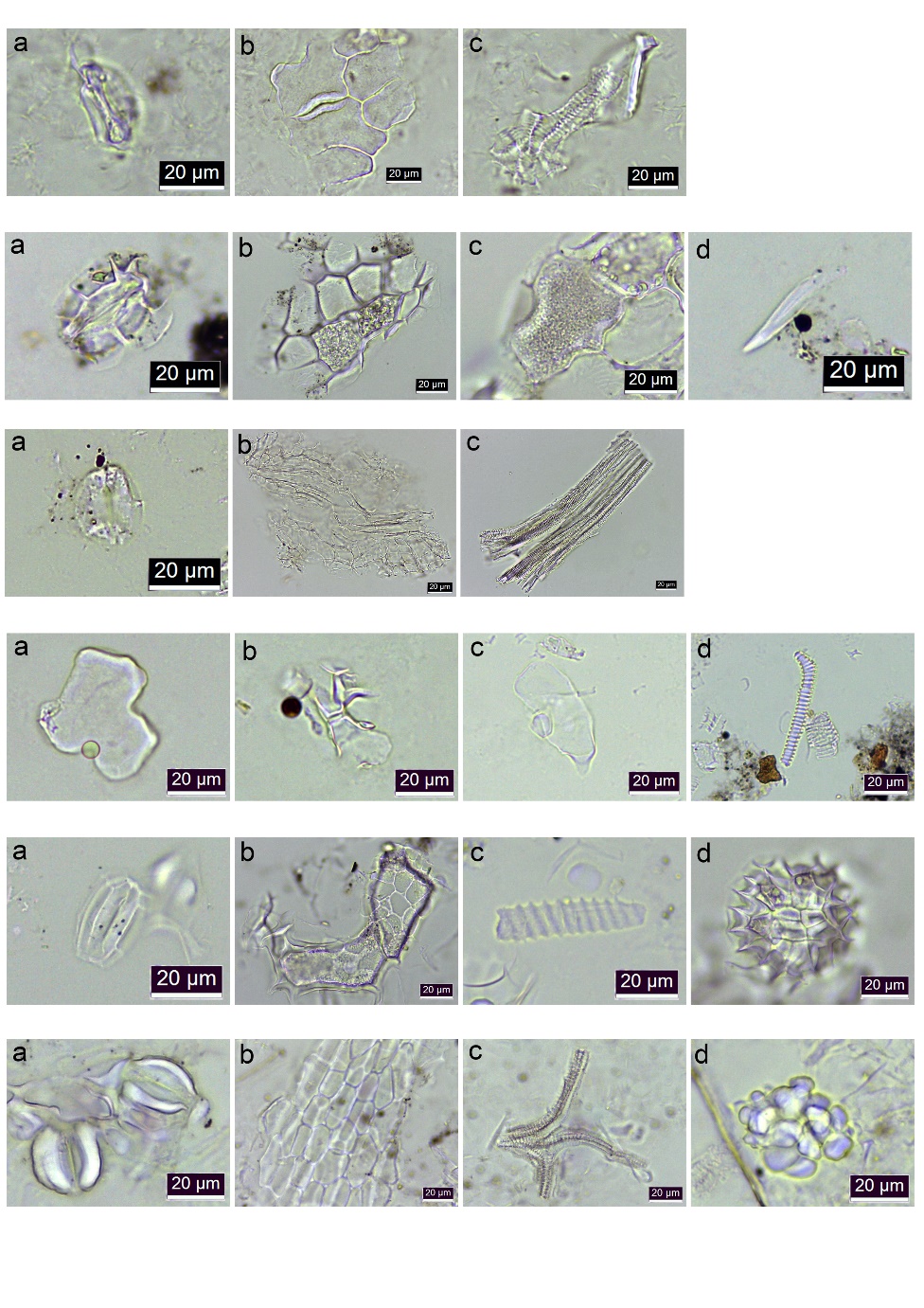


# Supplementary Figure 13.

I. *Salix ernestii*, a. articulated Stomate stellate and Polygonal tabular, b. Spheroid favose, c. Tracheary annulate.

II. *Philadelphus schrenkii*, a. Irregular sinuate, b. Spheroid favose, c. articulated Tracheary annulate and Spheroid favose.

III. *Paulownia fargesii*, a. Stomate stellate, b. Polygonal tabular and Hair base, c. articulated Spheroid favose, d. articulated Tracheary annulate.

IV. *Tilia mandshurica*, a. not fully silicified Polygonal tabular, b. Elongate entire extracted from twig, c. articulated Tracheary annulate.


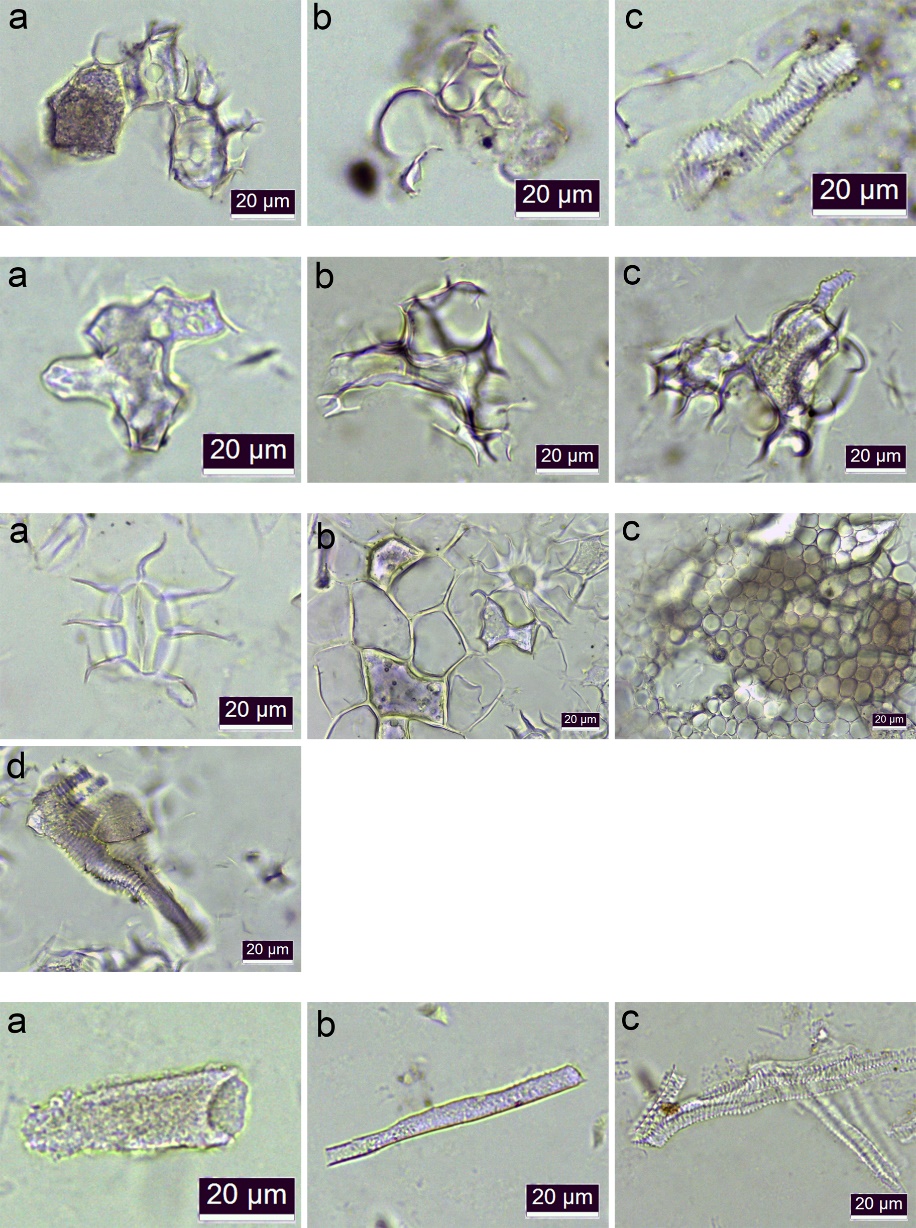

Supplement: Supplementary file 1 — Supplementary Information. [file 41598_2020_72547_MOESM1_ESM.docx]
